# A Universal Framework for Blood Ionome Extraction and Intelligent Quality Control in ^1^H NMR Metabolomics

**DOI:** 10.1021/acs.analchem.5c08255

**Published:** 2026-04-21

**Authors:** Anastasios Theodorou, Ivan Vučković, Eirini Papadimitriou, Konstantinos Papakonstantinou, Vasiliki Taki, Alexandra Louka, Costas Papaloukas, Constantine D. Stalikas, Nikolaos Giormezis, Fotini Paliogianni, Ian R. Lanza, Justin Stebbing, Panteleimon G. Takis

**Affiliations:** † Section of Analytical and Inorganic Chemistry, Department of Chemistry, 213703 University of Ioannina, Ioannina 45110, Greece; ‡ Department of Biological Applications and Technology, University of Ioannina, Ioannina 45110, Greece; § 6915 Metabolomics Core, Mayo Clinic, Rochester, Minnesota 55905, United States; ∥ School of Medicine and University Hospital, 37795 University of Patras, Rio 26504, Greece; ⊥ Department of Clinical and Experimental Epilepsy, 4919 Queen Square Institute of Neurology, University College London, London WC1N 3BG, U.K.; # Division of Endocrinology and Metabolism, Mayo Clinic College of Medicine, Rochester, Minnesota 55905, United States; ∇ School of Life Sciences, 2369 Anglia Ruskin University, East Road, Cambridge CB1 1PT, U.K.; ○ Department of Surgery and Cancer, Imperial College London, Hammersmith Hospital, Du Cane Road, London W12 0NN, U.K.; ◆ Section of Bioanalytical Chemistry, Division of Systems Medicine, Department of Metabolism, Digestion, and Reproduction, Imperial College London, Hammersmith Hospital, Du Cane Road, London W12 0NN, U.K.

## Abstract

Nuclear magnetic resonance (NMR) spectroscopy is a cornerstone of metabolomics and clinical bioanalysis; yet, its routine clinical adoption is limited by challenges in quality control (QC) and spectral interpretation. In blood-based NMR studies, ethylenediaminetetraacetic acid (EDTA) and its metal complexes are a major source of spectral interference, obscuring endogenous metabolites while increasing the analytical complexity. Here, we introduce a fully automated cheminformatics platform that enables EDTA-aware QC and the quantitative extraction of metal ion information while preserving metabolic integrity. Using large plasma and serum cohorts acquired at 500 and 600 MHz, we establish a multicenter-validated workflow for accurate quantification of calcium (Ca^2+^), magnesium (Mg^2+^), and zinc (Zn^2+^) directly from routine ^1^H NMR spectra (*R* ^2^ > 0.9 versus clinical and spectrometric assays). The platform harmonizes data sets across instruments and cohorts through automated baseline reconstruction. Application to a large breast cancer cohort reveals a stage-dependent decline in circulating Zn^2+^, consistent with disrupted zinc homeostasis in invasive disease. This work bridges metabolomics and metallomics, advancing the translational utility of NMR in precision medicine.

## Graphical abstract

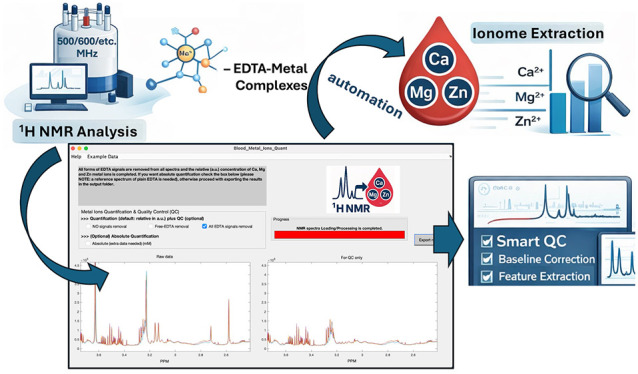

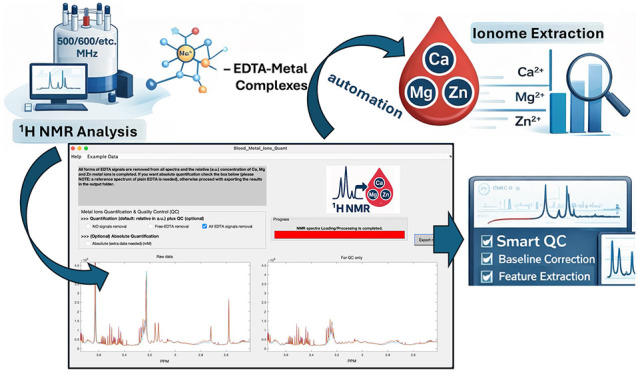

## Introduction

Nuclear magnetic resonance (NMR) spectroscopy is a robust, quantitative, and highly reproducible analytical technique that has become an indispensable technology for metabolomics and clinical bioanalysis.^1–7^ Its nondestructive nature, minimal sample preparation requirements, and ability to provide absolute quantification make it particularly well-suited for high-throughput biomarker discovery.^8,9^ Among biological fluids, blood-derived matrices such as serum and plasma are the most commonly analyzed in NMR-based metabolomics, owing to their accessibility and complementarity to standard clinical diagnostics. Over recent decades, various standard operating procedures (SOPs) have been established to guide sample handling, spectral acquisition, and data processing, with the goal of harmonizing workflows and improving interlaboratory reproducibility.^10,11^

Despite these efforts, a substantial portion of the literature still lacks explicit QC procedures, particularly those aimed at identifying and mitigating spectral artifacts in ^1^H NMR metabolic profiles.^12^ A major challenge is the complexity of QC workflows, which often require specialized NMR expertise to recognize and exclude signals arising from exogenous contaminants.^13^ These signals are sometimes erroneously removed due to misassignments or suboptimal signal suppression techniques, resulting in the unintended loss of biologically relevant information.

A prominent example is the widespread use of EDTA as an anticoagulant in plasma collection. EDTA introduces intense ^1^H NMR signals that frequently overlap with those of endogenous metabolites, thereby complicating spectral interpretation.^14^ The selective removal of EDTA and its metal complexes from NMR spectra remains a matter of debate. While the complete spectral characterization of all possible metal-ion EDTA complexes—particularly those involving metals present at concentrations above 10 μM (Figure S1), which is close to the detection limit of standard analytical NMR systems—remains incomplete, many existing protocols advocate for the exclusion of entire spectral regions dominated by Ca– and Mg–EDTA signals.^11,15^ However, this approach not only fails to eliminate all metal–EDTA signals but also leads to the unintended loss of coresonating endogenous metabolites. Despite these limitations, such region-based exclusions are widely adopted in untargeted metabolomics studies, particularly those relying on full-spectrum ^1^H NMR data for multivariate statistical analyses and biomarker discovery.^11^ Conversely, other studies recommend the selective removal of only free EDTA signals, arguing that metal–EDTA complexes may encode biologically meaningful information,^16,17^ especially in relation to metal ion homeostasis.

A few preliminary studies, primarily involving model aqueous systems or small numbers of real-world blood samples (*n* < 20), have shown that it is possible to recover accurate concentrations of added Ca^2+^, Mg,^2+^ and Zn^2+^ using NMR.^18–22^ Two studies employed photometric techniques to analyze a large number of blood samples to compare only Mg^2+^ measurements by NMR for a diabetic cohort, without providing any available algorithm to be adopted for widespread use and without any automation.^23,24^ To date, no large-scale investigation has been conducted using real-world blood samples to directly compare multiple metal–EDTA complexes measured by NMR with clinically measured metal ion concentrations, and there remains a lack of automated approaches that would facilitate the broader adoption of NMR by nonexpert users in clinical settings.

In addition, there is an urgent need for more accessible and standardized QC strategies, enabling clinicians and nonspecialists to perform plasma metabolomics with confidence. In this study, we introduce a fully automated platform that effectively removes NMR signals corresponding to free EDTA and its Ca–, Mg–, and Zn–EDTA complexes from EDTA-containing samples while minimizing the loss of signals from endogenous metabolites to maintain the metabolic profiles as intact as possible. More importantly, we demonstrate and rigorously validate that these metal–EDTA complexes exhibit strong correlations with clinical measurements of the corresponding ions, achieving close agreement in absolute concentrations across a large clinically profiled cohort, supplemented by a smaller cohort collected at an independent center and at a different magnetic field. Beyond improving QC workflows, our platform enhances the clinical relevance of NMR by enabling the investigation of mineral homeostasis through ^1^H NMR profiles. This advancement marks a significant step toward broader implementation of robust, high-quality NMR metabolomics in clinical research and diagnostics. Finally, we apply our platform to a large breast cancer cohort, revealing disruptions in zinc homeostasis that appear to be associated with an increase in tumor invasiveness.

## Experimental Section

### Serum–Plasma Sample Collection

Various multicenter serum/plasma cohorts were analyzed in this study (in total, ∼2000 samples were employed): 108 samples from the University Hospital of Patras (UoP) and 20 serum samples from the Mayo Clinic were used for the standardization and validation of the automated metal ions absolute quantification platform. The UoP samples were randomly selected from surplus material obtained from routine blood analyses of admitted patients (study protocol approved by the UoP Research Ethics Committee; protocol number 459/5-12-2024). The Mayo Clinic samples were obtained from the institutional biobank. In addition, 999 plasma-EDTA samples from the Breast Screening and Monitoring Study (BSMS) cohort were included, comprising women recalled from routine mammographic screening. The BSMS was approved by the Riverside Research Ethics Committee (Imperial College Healthcare NHS Trust; Tissue Bank Ethics/REC reference numbers: 12/LO/2019; 13/LO/1152; R10015-16A; 07/Q0401/20) and conducted in accordance with the Declaration of Helsinki and Good Clinical Practice guidelines. Finally, the ^1^H NMR spectra of 868 plasma-EDTA samples from the MTBLS147 study in the MetaboLights repository were employed to construct the chemical shift automated assignment part of our platform, where all samples were acquired using a Bruker spectrometer (Bruker BioSpin) operating at 600.13 MHz. All samples from all cohorts were stored at a temperature of −80 °C prior to analysis.

### Reagents and NMR Sample Preparation with and without EDTA

For this study, all of the reagents were purchased from Sigma-Aldrich. The buffer used for NMR sample preparation consisted of 75 mM Na_2_HPO_4_, 6.2 mM NaN_3_, and 4.6 mM sodium 3-(trimethylsilyl)­(2,2,3,3-*d*_4_)­propionate (TSP-*d*_4_) in H_2_O with 20% (v/v) deuterium oxide (D_2_O) at pH 7.4.

For metal ions standard curves and spikings, the following reagents were used: calcium chloride hexahydrate (CaCl_2_·6H_2_O), magnesium chloride hexahydrate (MgCl_2_·6H_2_O), and zinc sulfate heptahydrate (ZnSO_4_·7H_2_O), all of them with purity >98%. Additionally, metal ions were spiked into the buffer prior to sample addition to obtain a series of samples with different final concentrations for each ion.

Ethylenediaminetetraacetic acid (EDTA) tripotassium (3K) dihydrate (2H_2_O) was used for all of the EDTA experiments. EDTA was added to the NMR phosphate buffer at a concentration of 5.649 mM (i.e., ∼2.8 mmol/L of EDTA in the NMR sample), which is close to the concentration in common plasma collection tubes used in clinics when diluted by 50% in the NMR tube with the added serum sample. All plasma and serum NMR samples were prepared by adding 400 μL of the sample to 400 μL of the above-mentioned plasma buffer. The mixture was shaken gently (not vortexed), and a 600 μL aliquot was transferred to a 5 mm NMR tube.

### ^1^H NMR Spectra Acquisition

UoI ^1^H NMR spectra were recorded on a Bruker AV-500 NEO, equipped with a TXI cryoprobe at a ^1^H frequency of 500.13 MHz and an automatic cooled sample changer. The MAYO spectra were recorded using a Bruker IVDr 600 MHz spectrometer (Bruker BioSpin) equipped with a 5 mm BBI S3 (MC) probe and an automatic refrigerated sample changer (SampleJet). BSMS spectra were previously acquired at the National Phenome Centre at Imperial College of London using a Bruker IVDr 600 MHz spectrometer (Bruker BioSpin) equipped with a 5 mm PATXI H/C/N with 2H-decoupling probe including a *z*-axis gradient coil, an automatic tuning-matching (ATM), and an automatic refrigerated sample changer (SampleJet).

For all spectra, the sample temperature in the magnet was regulated to 310 ± 0.1 K by using a BTO variable temperature unit. The 1D ^1^H NMR spectra were recorded by suppressing the water peak using the standard 1D NOESY pulse sequence (noesygppr1d; Bruker BioSpin), acquiring 32 scans with 98,304 data points, an 18,029 Hz spectral width, a 10 ms mixing time, and a 4 s relaxation delay. A line broadening of 0.3 Hz was applied to the free induction decay before the Fourier transformation. Phase and baseline corrections were performed automatically via IconNMR (Bruker).

## Methods

### Independent Measurements of Metal Ions in Serum/Plasma Samples

It should first be noted that routine clinical analyses of common metal ions in blood are typically performed using serum samples, as these do not contain anticoagulants that could interfere with standard operating procedures (SOPs) for metal ion measurements (see below for further details), followed by clinicians/biochemists. Accordingly, to enable a fair comparison, our study primarily employed serum samples to which EDTA was added (see Experimental Section for details on NMR sample preparation), complemented by a smaller number of plasma-EDTA samples.

#### Clinical/Biochemistry Methods for Calcium and Magnesium from UoP and Mayo Clinic

Clinical measurements of calcium and magnesium in the UoP serum samples were performed according to the SOPs of the Alinity c analyzer reagent kits (Abbott). Briefly, calcium quantification was based on a colorimetric assay using the Arsenazo III dye reagent, while magnesium was determined spectrophotometrically via an enzymatic reaction involving isocitrate dehydrogenase and subsequent formation of NADPH.

For the Mayo Clinic samples, serum calcium and magnesium concentrations were measured using the COBAS c311 analyzer (Roche Diagnostics). Calcium was quantified through a two-step colorimetric method employing 5-nitro-5′-methyl-BAPTA (NM-BAPTA) and EDTA under alkaline conditions, whereas magnesium was determined via complex formation with xylidyl blue. In both laboratories, color intensity was proportional to analyte concentration, and all procedures were conducted in strict accordance with the manufacturers’ protocols.

#### Atomic Absorption Spectrometry (AAS) for Zinc

Stock standard solutions of Zn^2+^ (1000 ± 2 mg L^–1^ each) were purchased from Merck (Darmstadt, Germany). Suprapur grade HNO_3_ used in the experiments was also obtained from Merck (Darmstadt, Germany). Diluted solutions of each metal were prepared in 2% HNO_3_. Atomic absorption measurements were performed using a GBC AAS 932 Plus (Braeside, Australia). The AAS system was standardized, and data were evaluated using GBC Avanta software, version 2.01 (copyright 1996, GBC Scientific Equipment Pty, Ltd.). A single-element hollow-cathode lamp for Zn was sourced from Proton Pty. Ltd. (Victoria, Australia). Measurements for Zn were conducted at a wavelength of 213.9 nm, using a slit of 0.5 nm. A deuterium background corrector was used throughout all of the experiments. The resulting standard curve that was used for the zinc measurements in the 18 serum/plasma samples is depicted in Figure S2.

### Computational Details, Software, Chemical Shift Models Construction and Signals Deconvolution

For the present study, δ prediction models fitting and their statistics were performed by MATLAB 2024b (MathWorks), using the built-in functions *fitlm.m*. In particular, the assigned ^1^H NMR signals of the free EDTA, Ca–EDTA, Mg–EDTA, and Zn–EDTA from in total 906 serum/plasma EDTA ^1^H NMR spectra from multicentered cohorts were employed to construct the relationships between metabolites ^1^H NMR spin systems δ values, and their predicting ability validation was performed in large validation data set consisting of a total 1129 plasma-, serum-EDTA samples. Thirty out of the 1129 samples were spiked with Zn^2+^ to confirm the exact position of the Zn–EDTA NMR signals. All of the cohorts used for the study regarding chemical shifts automated assignment are summarized in Figure S3.

Part of the training and validation data sets of δ models are provided as Supporting Data set S1 and Data set S2 files.

For the ^1^H NMR signal deconvolution and QC component of our software, both Python and MATLAB components were combined to achieve the best deconvolution results.

A more detailed description of the whole construction of the developed chemometrics software can be found in Supporting Information section: *Analysis of the algorithm construction* – *computational and functional details*.

In addition to the above-mentioned programming suites, extra statistical software was employed (i.e., GraphPad Prism 10) for performing routine ANOVA, *F*-tests, and other statistical analyses, whereas the MATLAB-based PLS_Toolbox v9.2.1 (Eigenvector Research, Inc., Manson, WA 98831; software available at http://www.eigenvector.com) was used for multivariate statistics (MVA analyses). Before PCA analyses, the spectra were binned (i.e., bin-width of 0.02 ppm) and the resulting data sets were mean-centered.

### Integrals Normalization for Absolute Ion Quantification from Multicohorts

As previously mentioned, the automated absolute quantification of the metal ions was validated with two multicenter cohorts, where the spectra were acquired on two different NMR spectrometers (500 MHz: UoI cohort and 600 MHz: Mayo cohort) that were clinically analyzed at two different sites (UoP and Mayo). The samples employed for the construction (i.e., production of standard curves for each metal ion) and validation of our algorithm for the automated (absolute/relative) quantification of metal ions are summarized in Figure S4.

To enable direct comparison and integration of data acquired at different magnetic field strengths (500 and 600 MHz), a universal calibration curve was established for each metal ion (Ca^2+^, Mg^2+^, and Zn^2+^). Standard solutions containing K_3_-EDTA in water/plasma buffer were prepared following the SOPs for serum and plasma NMR metabolomics.

The instrument sensitivity (*S*_*f*_) for each spectrometer operating at field *f* was calculated from the integral of a reference free EDTA peak (Int_EDTA,peak1,*f*_) and the corresponding spectral noise (σ_*f*_):

1 $$S_{f}=\frac{{\mathrm{Int}}_{\mathrm{EDTA},\mathrm{peak}1,f}}{\sigma_{f}}$$

In our case, σ_*f*_ values were calculated by the standard deviation of the 13.0–14.0 ppm of the ^1^H NMR spectral region.

The normalized integral of each metal–EDTA complex (*I*_*M*,*f*_^norm^) was then computed as

2 $$I_{M,f}^{\mathrm{norm}}=\frac{{\mathrm{Int}}_{M\mathrm{-EDTA},f}}{{\mathrm{Int}}_{\mathrm{EDTA},\mathrm{peak}1,f}}\times \frac{S_{\mathrm{ref}}}{S_{f}}$$

where *S*_ref_ denotes the reference sensitivity (e.g., 500 MHz).

These normalized integrals were used to construct universal calibration curves for each ion according to

3 $$C_{M}=a_{M}I_{M,f}^{\mathrm{norm}}+b_{M}$$

where *C*_*M*_ is the absolute ion concentration and *a*_*M*_ and *b*_*M*_ are the fitted slope and intercept parameters. This approach ensures cross-instrument normalization and accurate absolute quantification of metal ions across multicenter data sets. It is noted that assessment of the 95% confidence intervals of the calibration curves indicated that, although a nonzero intercept was obtained from the unconstrained fits (see Supporting Information section: *Metal ions standard curves for the 500 MHz*), the curve passes statistically through the origin. However, the presence of a nonzero intercept reflects residual errors from spectral noise and uncertainties of the NMR signals fitted by our algorithm.

By employing the above strategy, provided that all NMR spectra are acquired with the same pulse sequence and NMR parameters [i.e., at least the same *receiver gain-RG* values (even though our algorithm accounts for RG normalization), *number of scans-ns*, *time delay–D1* and *similar 90*° *pulse duration-P1*], we are able to get the absolute quantification automatically via our algorithm, regardless of the magnetic field of the employed NMR spectrometer (i.e., 500 or 600 MHz).

## Results

### Automated Assignment of Free and Metal-Bound EDTA Complexes in ^1^H NMR Profiles

Building on our previously established strategies for predicting chemical shifts (δ),^25–27^ we developed mathematical models correlating δ values of metabolites commonly found in blood—specifically those that are highly abundant and frequently observed—with the NMR signals of EDTA and its predominant metal complexes: Ca–EDTA, Mg–EDTA, and Zn–EDTA (Figure S1). To achieve this, we used two cohorts comprising a total of 936 one-dimensional (1D) ^1^H NMR spectra: 868 from plasma-EDTA^28^ and 68 from serum-EDTA samples acquired internally and spiked with Zn^2+^ to ensure accurate assignment of Zn–EDTA signals. All NMR samples were prepared and analyzed according to standardized operating procedures (SOPs),^11^ as detailed in the Experimental section.

Signals corresponding to Ca–EDTA and Mg–EDTA were consistently detected across all of the spectra. Due to the low endogenous abundance of Zn^2+^ (Figure S1), Zn–EDTA signals were further resolved by spiking the 68 serum-EDTA samples with Zn^2+^, enabling unambiguous assignment of Zn–EDTA δ spin systems. These 68 spectra were randomly divided into a training set (*n* = 38) and a validation set (*n* = 30) to ensure robust model construction and evaluation. The δ value ranges are shown in Figure S5, highlighting substantial variability of the metabolic content—exceeding the second decimal place of the ppm scale—across all studied signals in the training data sets. The observed δ variability reflects the highly variable physicochemical environment that is experienced by proton nuclei of the metabolites in our samples, such as variable concentrations of metabolites, inorganic ions, pH, temperature, and numerous weak intermolecular interactions (e.g., hydrogen bonding, electrostatic effects, and transient complex formation). As previously shown by various studies,^25,29^ in multicomponent biological mixtures, these interactions collectively modify the local electronic environment, so that chemical shifts are not fixed constants but continuous functions of the overall sample composition.^25,29^ As illustrated in Figures 1A and S6, the δ value of the anomeric proton of glucose shows a linear correlation with both the Ca–EDTA quartet and the Zn–EDTA singlet/quartet. Based on the training data, the corresponding fitted models (Figure 1B,D) exhibit *R* ^2^ values exceeding 0.97, indicating that prior knowledge of the glucose doublet δ enables highly accurate prediction of the corresponding δ values for the specific spin systems from the Ca– and Zn–EDTA complexes. Notably, the calculated relative root mean squared error (rRMSE) values for the models remain well below the typical NMR line width threshold (< ± 1 line width), underscoring models’ precision (Figure 1B,D). Accordingly, we developed three additional δ models to predict the remaining proton spin system δ values for each of the three metal–EDTA complexes (Figures 1C,E, and S6). All models exhibited strong linearity and high predictive accuracy, with *R* ^2^ values exceeding 0.98. The predicted δ values consistently fell within a margin of less than two line widths, demonstrating the robustness and precision of these models. All model predictors (*x*-values) and response variables (*y*-values) are indicated by black arrows in Figures 1A and S6, with the corresponding statistical metrics summarized in Table S1. Based on these models, a comprehensive chemical shift prediction map (Figures 1 and S6) supports the conclusion that assignment of all ^1^H NMR signals corresponding to the three most abundant metal–EDTA complexes can be fully automated.

**Figure 1:**
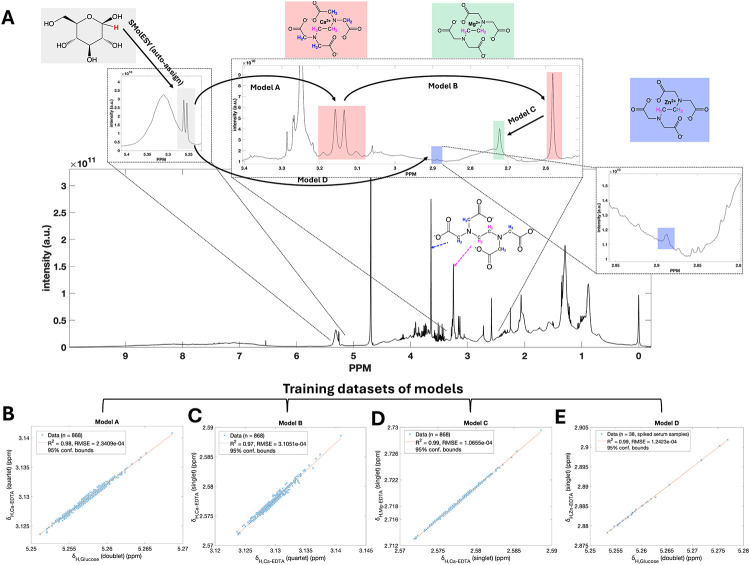
Predictive models of ^1^H NMR chemical shifts (δ) for free EDTA and EDTA–metal complexes, and the combinatorial strategy used for their integration. (A) The δ values of the anomeric ^1^H of glucose (red font) were linearly correlated with (B) the quartet (i.e., AB quartet) δ values of Ca–EDTA protons (blue font) (*R* ^2^ = 0.98; model A), which in turn correlated with (C) the Ca–EDTA singlet δ values (magenta font) (*R* ^2^ = 0.97; model B). (D) The Ca–EDTA singlet δ values showed a strong linear relationship with those of Mg–EDTA (magenta font) (*R* ^2^ = 0.99; model C). (E) For Zn–EDTA, both spin systems exhibited near-perfect linear correlations with the anomeric ^1^H NMR δ values of glucose (*R* ^2^ = 0.99). As illustrated here and in Figure S6, glucose—which is ubiquitously present and readily detectable in blood by ^1^H NMR—serves as a reliable internal reference, enabling the accurate prediction of δ values for all major EDTA–metal complexes. All predictive δ models were trained using 868 plasma-EDTA spectra from the MetaboLights repository (MTBLS147)^28^ and 38 serum-EDTA sample spectra spiked with zinc. Detailed information on data set composition and model construction is provided in the Methods section (“*Computational details, Software, construction of chemical shift models, and signals deconvolution*”). Some examples of the rest of the models and summary statistics are described in Figure S6 and Table S1.

Given that glucose is sufficiently present in >99% of human serum and plasma samples—even under pathological conditions at concentrations above 100 μM—its anomeric proton signal (a characteristic doublet)^30^ can be reliably and automatically identified using SMolESY (Small Molecule Enhancement Spectroscopy) and *J*-coupling constants criteria in standard 1D ^1^H NMR spectra (Figure 2A).^27,31^ This provides a robust anchor point for accurately predicting and assigning the remaining δ values of the metal–EDTA complexes.

**Figure 2:**
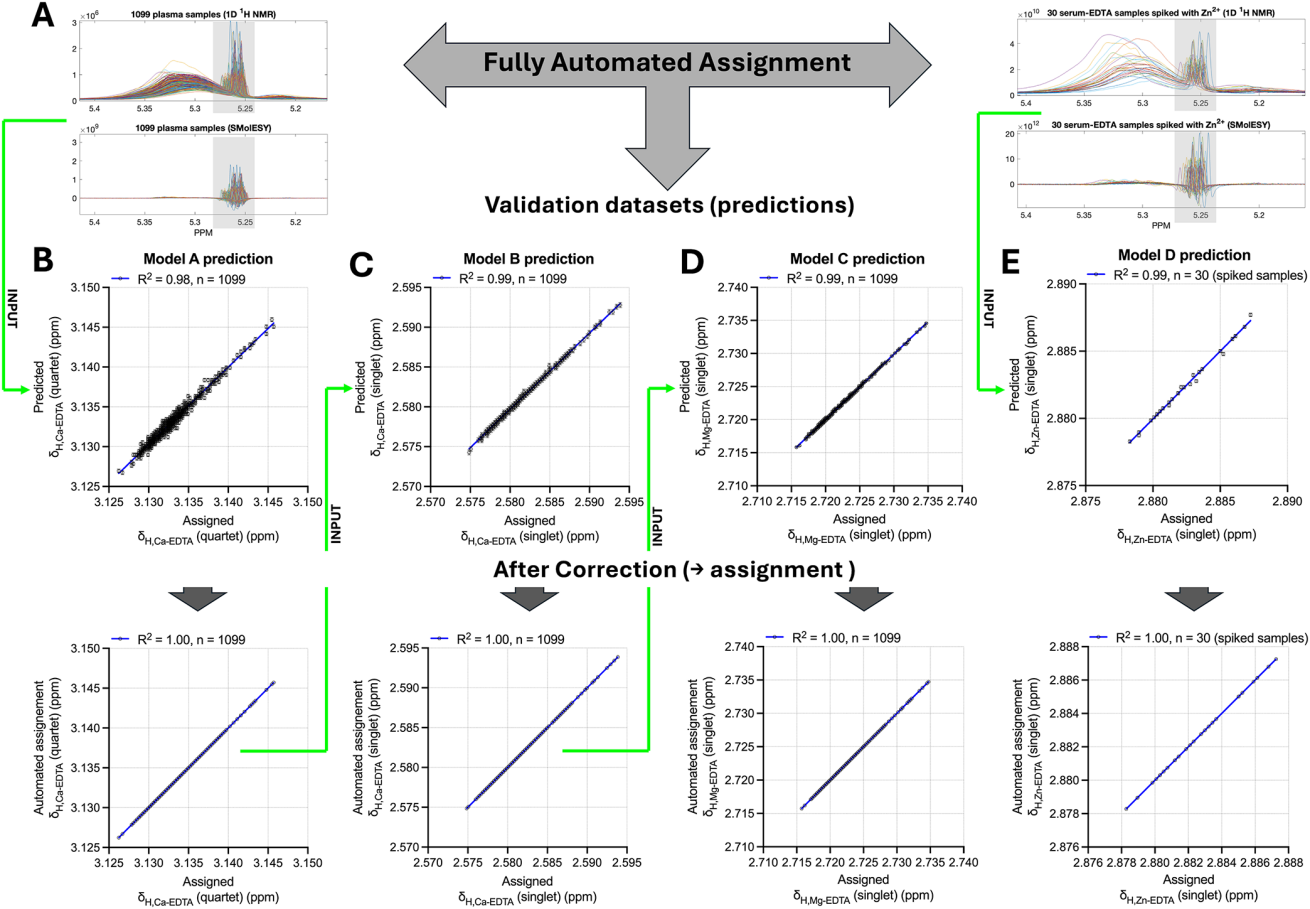
Validation of the δ prediction algorithm for ^1^H NMR signal assignment of major EDTA forms. (A) The algorithm first automatically assigns the δ values of glucose anomeric protons using SMolESY,^31^ and (B, upper panel) predicts the quartet δ values of Ca–EDTA via model A. Based on the model’s rRMSE values (B, lower panel), the algorithm autoassigns the corresponding quartet peaks, validated using an independent plasma-EDTA cohort (*n* = 1099; BSMS study).^32^ (C) The assigned Ca–EDTA quartet δ values are then used as inputs (green arrows) for prediction of the Ca–EDTA singlet δ values via model B (upper panel), which are subsequently confirmed through automated maxima detection (lower panel). (D) The resulting Ca–EDTA assignments are employed to predict Mg–EDTA δ values using model C (upper panel), followed by accurate assignment in the validation cohort (lower panel). (E) For Zn–EDTA signal assignment, 38 serum-EDTA samples spiked with zinc were analyzed to validate δ value predictions derived from glucose (model D, upper panel) and their automated assignment (lower panel). Additional model details are provided in Figure S6.

Building on this principle, we developed a fully automated algorithm that begins by assigning the glucose anomeric proton δ (Figure 2A). It then sequentially applies the trained δ models, incorporating each model’s prediction error as quantified in absolute values by relative RMSE (rRMSE) (Table S1) to accurately assign the remaining metal–EDTA signals. Namely, the predicted δ values together with the rRMSE define an infinitesimal search window (i.e., less than 2 line widths wide), within which the algorithm identifies the observed local maximum that corresponds to the true peak of interest. Therefore, each step of the process is anchored to an actual spectral feature rather than relying only on the predicted value, prohibiting error propagation. The selected sequence of δ models (Figures 1A, S6A) was based on their performance (Table S1). If a model underperforms—for example, Model B (Figure 2)—the algorithm applies the next most reliable δ model, typically based on the assigned δ value of the glucose anomeric proton model, which shows a slightly higher rRMSE value (i.e., the search window becomes 2.5 line widths wide).

To validate the algorithm, we applied it to an external cohort consisting of 1099 plasma-EDTA ^1^H NMR spectra^32^ and the independent set of 30 Zn^2+^-spiked serum-EDTA spectra, as detailed in the Methods section. The performance of the algorithm across these large validation data sets for the majority of the peaks is illustrated in Figure 2B–E. Notably, the δ predictions for each spin system fell within a narrow margin of error—consistently within two NMR line widths (Figure 2B–E, upper panels)—enabling precise and automated assignment of the ^1^H NMR signals associated with each metal–EDTA complex, as shown in the lower panels of Figure 2B–E.

Through this validation, we demonstrate the successful and robust implementation of a fully automated pipeline for the assignment of all ^1^H NMR signals arising from the most abundant metal–EDTA complexes in blood and serum. Remarkably, this process requires only the raw 1D ^1^H NMR data as input. To the best of our knowledge, this level of automation has not been previously achieved. Its significance lies not only in enabling the automated exclusion of these occasionally confounding signals during NMR-based plasma-EDTA profiling but also in offering a streamlined method for the quantification of metal ions (particularly for low-abundance species such as Zn^2+^) with minimal risk of misassignment. Parts of various δ models training and validation data sets are provided in the Supporting Data set S1 and Data set S2 files, respectively.

### Quantification (Relative/Absolute) of Metal Ions in Blood vs Clinical/Spectrometric Values

The automated assignment of ^1^H NMR signals arising from calcium–, magnesium–, and zinc–EDTA complexes enables the reliable quantification of these metal ions in plasma and serum samples. Although a few previous studies have employed metal–EDTA complexes for the quantification of biologically relevant metal ions using NMR spectroscopy, these approaches have generally lacked extensive validation, automation, and standardization—factors essential for integration into high-throughput clinical workflows, large-scale omics platforms, and biomarker discovery pipelines. Therefore, we sought to automate this process and extensively validate it to standardize metal ion quantification in human biofluids (i.e., blood biospecimens), offering a robust and scalable solution for routine metal ion profiling using only raw 1D ^1^H NMR data.

To enable the quantification of metal–ions, we modeled the distinct ^1^H NMR spin system patterns of each metal–EDTA complex using reference spectra acquired in aqueous solution, prepared under conditions that replicate those of plasma and serum NMR samples (see Methods section). The ^1^H NMR fingerprints were modeled by *Voigt* functions (i.e., a combination of *Lorentzian* and *Gaussian* functions) and were stored in a database that is used for the automated fitting of the corresponding signals in real-world samples, implemented in our developed pipeline (see the Supporting Information: *Analysis of the algorithm construction – computational and functional details*). Since free EDTA, calcium, and magnesium are highly abundant (Figure S1), their NMR fingerprint can be straightforwardly fitted and deconvoluted via their modeled patterns as shown in Figures 3A,B, and S7. This process is greatly facilitated by the ability to automatically assign the chemical shifts of these spin systems, as demonstrated by extensive validation in a large cohort of plasma-EDTA spectra. For Zn–EDTA, however, its low abundance and the overlap of its NMR signals with those of other metabolites—primarily the doublet of doublets from the asparagine −CH_2_ protons—necessitated an additional deconvolution step. Specifically, the modeled peaks of asparagine were first removed (Figure 3C).

**Figure 3:**
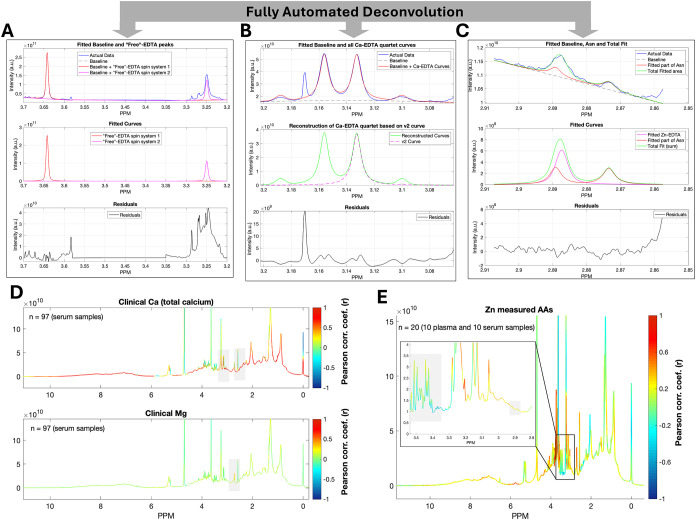
Automated deconvolution and validation of free EDTA and EDTA–metal complex signals in serum ^1^H NMR profiles. Representative deconvoluted signals are shown for (A) nonchelated EDTA and its metal complexes, including (B) Ca–EDTA and (C) Zn–EDTA, in serum-EDTA ^1^H NMR profiles. The deconvolution process is fully automated, facilitated by Voigt-function modeling of each EDTA form and their precise δ values assignment obtained through our algorithm. Among these, deconvolution of the Zn–EDTA singlet presents a particular challenge due to the low abundance of zinc and signal overlap with the asparagine spin system. Nevertheless, the algorithm successfully resolves these signals owing to the highly accurate δ value predictions (Figure 2E). Validation of metal ions’ presence in the ^1^H NMR profiles via their chelation with EDTA was performed through correlation analysis of NMR-derived integrals with independent clinical/spectrometric measurements of metal ions. (D) Pearson correlation plots show strong agreement (*r* > 0.95) between Ca–EDTA NMR signals and clinically measured concentrations of Ca (upper panel) as well as for Mg (lower panel) across 97 serum-EDTA samples. (E) Zn–EDTA signals, evaluated against atomic absorption spectrometry (AAS) measurements in 20 samples (10 plasma and 10 serum), also demonstrated positive correlations (*r* ≈ 0.7–0.8), consistent with zinc’s lower abundance in blood. Notably, Ca levels exhibited a high correlation with total protein concentration, reflecting albumin binding effects.^33^ Further methodological details are provided in the Methods section (*“Independent measurements of metal ions in serum/plasma samples”*).

Quantification was carried out in two stages: relative and absolute. In the relative stage, the deconvolution process provided the relative concentrations of the ions based upon their EDTA complex ^1^H NMR singlets. Validation of the relative quantification was performed by comparison to independently determined concentrations of Ca^2+^, Mg^2+^, and Zn^2+^ obtained through established clinical and laboratory methodologies. Calcium and magnesium were quantified in 97 serum samples using routine clinical protocols at the UoP site, while zinc concentrations were measured in 18 blood samples (8 serum and 10 plasma-EDTA) by atomic absorption spectrometry (AAS) (see Methods section for additional details of clinical and AAS measurements). The integrals of the deconvoluted NMR peaks (i.e., in our case from the ^1^H NMR singlets of each metal–EDTA complex) exhibited strong linear correlations with the corresponding absolute concentrations (*R* ^2^ > 0.90; Figure S8).

Correlation analysis of the ∼131,000 spectral features derived from the 97 serum-EDTA spectra further confirmed these findings. Pearson correlation coefficients (*r*) approached unity for the Ca–EDTA ^1^H NMR signals (Figure 3D, upper panel). Albumin-associated features also displayed elevated correlations, reflecting the known contribution of albumin to the total circulating calcium. Analogous analyses demonstrated similarly strong correlations for Mg–EDTA signals with clinically determined magnesium concentrations (Figure 3D, bottom panel), while subsets of Zn–EDTA features correlated well with AAS-derived zinc values (Figure 3E). Collectively, these results establish that automated deconvolution of metal-ion EDTA signals yields quantitative estimates, consistent with independent clinical and laboratory measurements.

Building on this validation, we next sought to determine the absolute concentrations of metal ions directly from the NMR spectra. Calibration curves were generated for Ca^2+^, Mg^2+^, and Zn^2+^ (Figure S9A–C) across physiologically relevant ranges reported in the Human Metabolome Database (HMDB).^34,35^ The deconvoluted integrals of the metal-ion EDTA singlets were then employed to derive absolute concentration values, thereby enabling the direct translation of NMR spectral features into clinically meaningful readouts.

In the first application of this approach, automated ion quantification of 115 blood spectra (97 from the UoP cohort and 18 additional samples; acquired at 500 MHz, see Experimental Section) demonstrated excellent agreement with clinical measurements of Ca^2+^ and Mg^2+^, as well as AAS-derived Zn^2+^ concentrations (Figure S10A–C). To further establish the robustness and generalizability of the method, validation was extended to an independent external cohort of serum-EDTA spectra acquired at the Mayo Clinic (600 MHz; prepared as indicated in the Experimental section). As these data sets originated from instruments operating at different ^1^H NMR Larmor frequencies (i.e., 500 and 600 MHz), calibration curves were reconstructed using normalized integrals of the metal–EDTA NMR signals. Normalization was achieved by calculating the ratio of the deconvoluted integrals of each metal–EDTA complex to those of free EDTA acquired at each field in the absence of metal ions (Figure 4A–C, upper panels). This enabled direct comparability across cohorts and quantification of metal ions, regardless of the magnetic field strength. Accordingly, upon deconvolution of the signals from both data sets, all integrals were normalized to the reference integrals from free EDTA peaks and applied to the recalibrated curves, allowing consistent and accurate quantification of absolute metal ion concentrations across multicenter cohorts and acquisition platforms (see Methods section: *Normalized integrals method for merging multicentered cohorts*). Linear regression analyses of NMR-derived versus clinically measured concentrations demonstrated strong correlations (*R* ^2^> 0.9) in the UoP cohort and moderately high correlations in the Mayo cohort, the latter reflecting the narrower dynamic range of metal ion concentrations in this data set.

**Figure 4:**
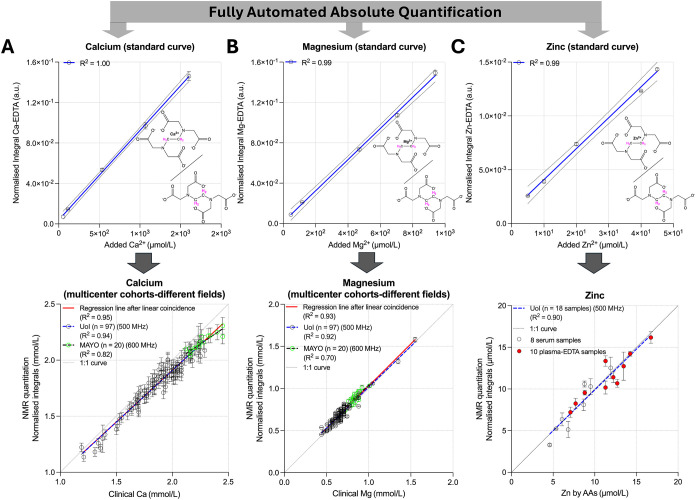
Construction and validation of standard curves for absolute quantification of EDTA–metal complexes by NMR independently of the magnetic field. Standard calibration curves were generated for (A) calcium (upper panel), (B) magnesium (upper panel), and (C) zinc (upper panel) complexes with EDTA in water/plasma buffer solutions prepared analogously to serum/plasma NMR samples. The curves were constructed from ^1^H NMR signal integrals of each metal–EDTA complex ^1^H NMR singlets, normalized to the corresponding integrals of free EDTA and adjusted for instrument sensitivity (see Methods section: *“Normalization for ion absolute quantification across multicohort data”*). The derived calibration functions were implemented into the algorithm and validated using two multicenter serum-EDTA cohorts comprising 97 (open black circles) and 20 (open green circles) serum-EDTA samples acquired at 500 MHz (UoI) and 600 MHz (Mayo), respectively, for (A) calcium (bottom panel) and (B) magnesium (bottom panel). (C) Validation for zinc was performed in an independent cohort of 18 samples (10 plasma-EDTA and 8 serum-EDTA) and benchmarked against atomic absorption spectrometry (AAS) data. The NMR-based absolute quantifications showed excellent agreement with independent measurements, yielding *R* ^2^ = 0.95 for calcium, *R* ^2^ = 0.93 for magnesium, and *R* ^2^ = 0.90 for zinc. Error bars for all plots are calculated from the peak-fitting residuals via our algorithm. These results confirm the accuracy and cross-instrument consistency of the developed method for metal ion quantification across multicenter cohorts.

Importantly, ANOVA (F-test) analysis of regression coincidence confirmed a near-perfect alignment between the regression curves of the two cohorts and the theoretical 1:1 line, underscoring the robustness and cross-cohort reproducibility of the method (Figure 4A–C, bottom panels, and Table S2). Additionally, within our algorithm, along with the limit of detection (LOD) and quantification (LOQ), the recovery percentage of metal ion concentrations was assessed through spiking experiments performed on a pooled serum-EDTA sample generated from the 97 individual serum spectra (Figure S11 and Table S3). These findings demonstrate that the algorithm enables the accurate, automated determination of absolute concentrations of EDTA-chelated Ca^2+^, Mg^2+^, and Zn^2+^ in serum and plasma, independent of magnetic field strength or acquisition site and in close agreement with established clinical and independent spectroscopic measurements. Importantly, the automation of this procedure substantially accelerates and standardizes the extraction of clinically relevant data directly from routine 1D ^1^H NMR spectra. To our knowledge, this represents the first fully automated, multicenter-validated workflow for absolute quantification of metal ions from EDTA complexes in blood, providing a readily implementable tool for integration into large-scale metabolomics and multiomics studies.

### Removal of ^1^H NMR Signals from “Contaminants” – Smart QC

Since the automated assignment of free EDTA and metal–EDTA complex signals and their deconvolution are incorporated into our workflow, the algorithm also enables the automated removal of unwanted ^1^H NMR signals arising from EDTA and its complexes when present in blood spectra (Figure 5A). This functionality was implemented by replacing the removed signal regions with baseline interpolation (see the Supporting Information: *Analysis of the algorithm construction – computational and functional details*) and was evaluated using 20 serum-EDTA samples. Specifically, we compared three approaches (Figure 5A): (i) automated removal of deconvoluted EDTA-related signals, (ii) manual removal of EDTA-associated peaks by selective “chopping” of NMR regions performed by an experienced spectroscopist, and (iii) spectra from matched serum samples without EDTA. To assess performance, principal component analysis (PCA) was applied to determine, in a statistically unsupervised and untargeted approach, which method of signal removal best preserved the underlying metabolic profiles (Figure 5B). The results showed that automated removal via deconvolution retained a higher degree of similarity to the original EDTA-free spectra compared to manual processing, thereby preserving more of the intrinsic metabolic information.

**Figure 5:**
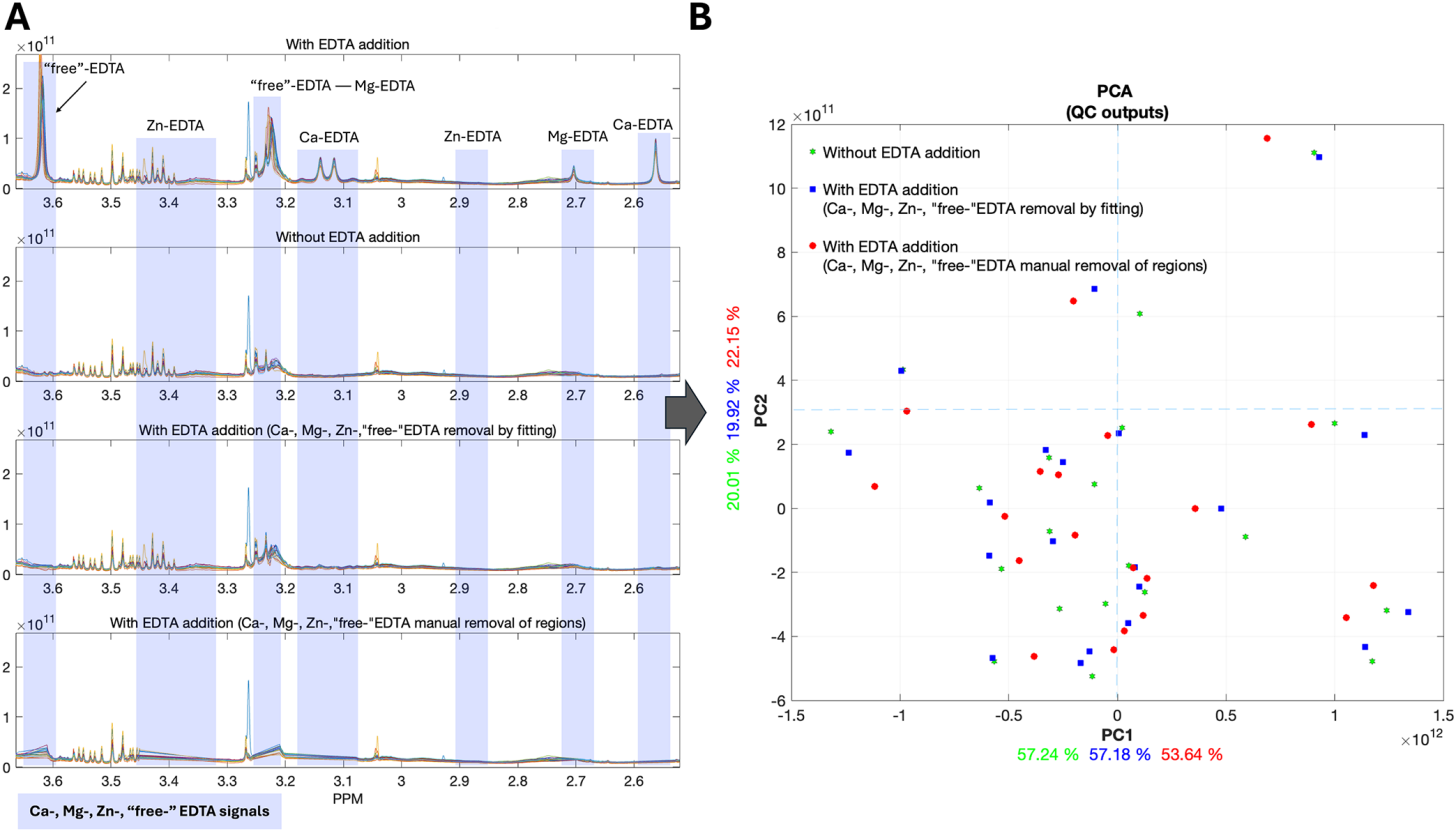
Improving NMR quality control (QC) of EDTA-containing blood samples. (A) Spectral regions from 20 serum ^1^H NMR spectra (focusing on the 2.5–3.7 ppm spectral region) are shown, encompassing all signals from free EDTA, Ca–EDTA, Mg–EDTA, and Zn–EDTA complexes (highlighted by the vertical boxes). The top panel displays spectra from serum-EDTA samples, followed by spectra from serum samples without EDTA (second panel). The third panel shows spectra after automated removal of all EDTA-associated signals using our QC algorithm, which performs automated assignment, signal fitting, and baseline interpolation after signal removal. The bottom panel presents spectra where EDTA-related signals were manually removed by cropping the corresponding spectral regions. (B) Principal component analysis (PCA) score plots of the 20 serum samples demonstrate the impact of the different preprocessing approaches. The cumulative variance explained by the first two PCs after automated EDTA signal removal (77.1%) closely matches that of noncontaining EDTA serum spectra (77.25%), indicating preservation of the underlying metabolic information. In contrast, manual removal of EDTA signals leads to greater information loss, reflected by a lower cumulative variance (75.79%). These results confirm that the automated QC algorithm effectively eliminates EDTA-related artifacts while minimizing metabolic information loss.

We have previously demonstrated and rigorously validated that signals arising from Ca–, Mg–, and Zn–EDTA complexes can be exploited for the accurate quantification of the corresponding metal ions in serum and plasma spectra. Although these signals provide valuable quantitative information, their presence in untargeted metabolomics analyses can be problematic, particularly when the analytical focus is on metabolites that resonate in close spectral proximity to the metal–EDTA complexes. In such cases, the selective removal of these signals is desirable. By contrast, the removal of free EDTA signals is necessary in all contexts, as they do not carry meaningful biochemical information and may confound downstream analyses.

To address these challenges, we incorporated into our algorithm a flexible functionality that enables users to automatically remove either free EDTA signals alone or both free EDTA and the dominant metal–EDTA signals. The removed regions are reconstructed by baseline interpolation, preserving the integrity of the surrounding spectral features. This automated procedure improves quality control by systematically reducing artifacts and ensuring consistency across large data sets. Importantly, our evaluation demonstrates that this approach minimizes the loss of genuine metabolic content while facilitating the streamlined preparation of data sets for high-throughput analyses. In particular, it produces metabolomics-ready spectral data sets that can be directly integrated into large-scale, automated omics pipelines without requiring manual preprocessing (see the Supporting Information: *Analysis of the algorithm construction – computational and functional details*).

### Automated Software Translation in Omics Data Sets – BSMS (Breast Cancer) Cohort

Having established and validated our algorithm for the automated identification and quantification of dominant metal ions via their EDTA complexes, we next applied it to a large breast cancer cohort previously analyzed by our group, in which correlations between cfDNA and metabolomics data had been identified in plasma-EDTA samples. In total, 1099 plasma-EDTA ^1^H NMR spectra were processed to quantify circulating Ca^2+^, Mg^2+^, and Zn^2+^ concentrations.^32^ Motivated by accumulating evidence that disrupted metal ion homeostasis contributes to cancer biology,^36^ we investigated whether such alterations could be detected in blood and whether they varied across breast cancer progression. Four clinical groups were examined: cancer-free controls, benign lesions, in situ carcinoma, and invasive breast cancer.

The algorithm successfully processed all spectra, and quality control analysis confirmed the robustness of the quantification, with pooled study samples clustering tightly near the [0,0] origin in the PCA plot (Figure 6A), consistent with technical reliability. Univariate analysis was then performed across the clinical groups using one-way ANOVA with false discovery rate (FDR) correction for multiple testing. Calcium and magnesium concentrations did not differ significantly across groups (Figure 6B,C). In contrast, zinc exhibited a stage-dependent decline, with significantly reduced plasma Zn^2+^ concentrations observed in patients with invasive breast cancer compared to those in cancer-free individuals or those with benign lesions, while in situ carcinoma displayed an intermediate trend (Figure 6D). Notably, only a weak correlation was observed between the Mg^2+^ concentration and body mass index (BMI), while none of the measured ions were statistically significantly correlated with the age of the enrolled individuals in the BSMS study. These findings reinforce that the observed decrease in the plasma Zn^2+^ concentration is specifically associated with increasing breast cancer severity (Figure S12).

**Figure 6:**
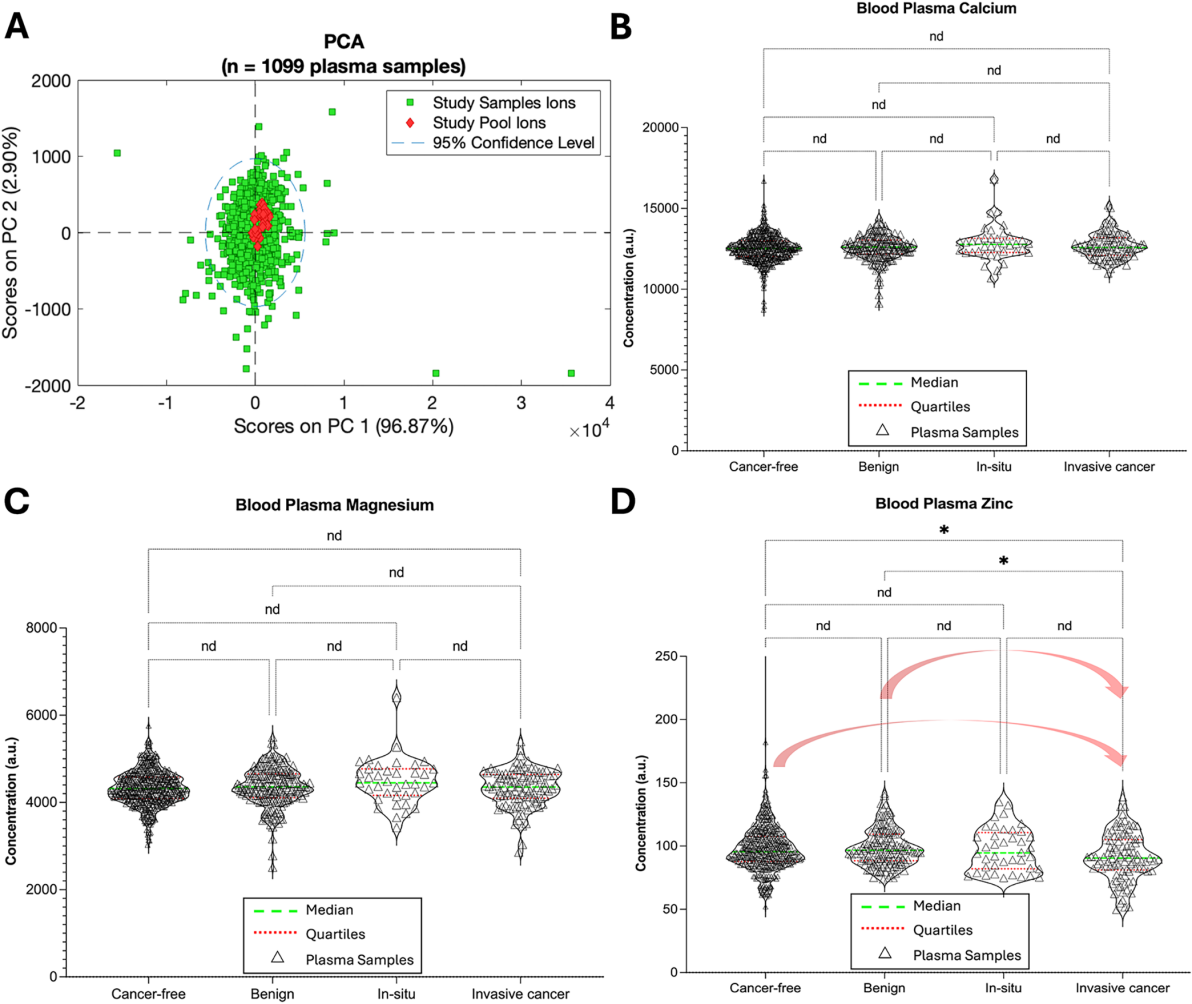
Application of our algorithm to the quantification of metal ions in plasma-EDTA samples from the BSMS cohort. (A) PCA score plot of the quantification results for Ca, Mg, and Zn obtained from 1099 plasma-EDTA spectra (comprising 100 study pool and 999 individual study samples) demonstrates excellent technical consistency, with the pooled QC samples clustering tightly around the [0,0] origin. Ordinary one-way ANOVA was performed for each automatically quantified metal ion (distribution of ions concentration is depicted via violin plots)—(B) plasma Ca, (C) plasma Mg, and (D) plasma Zn—across four clinical groups: cancer-free controls (*n* = 614), benign breast tumors (*n* = 215), in situ carcinoma (*n* = 40), and invasive breast cancer (*n* = 105). It should be noted that, because plasma zinc concentrations are substantially lower than those of calcium and magnesium (Figure S1), zinc concentrations used for the ANOVA analysis (panel D) were recovered from approximately 94% of the total samples. Specifically, the following sample numbers were included: cancer-free controls (*n* = 588), benign breast tumors (*n* = 193), in situ carcinoma (*n* = 40), and invasive breast cancer (*n* = 93). After correction for multiple testing using the false discovery rate (FDR) method (where asterisks denote significant discoveries, *q* < 0.05, and “nd” indicates nonsignificant results, *q* ≥ 0.05), only Zn exhibited a statistically significant decrease in concentration with increasing disease severity.

Strikingly, these findings mirror recently published studies implicating zinc as both a biomarker and a mechanistic contributor to breast cancer progression,^36,37^ thereby reinforcing the clinical and biological significance of our observations. To the best of our knowledge, this represents the first demonstration of fully automated, NMR-based quantification of circulating metal ion homeostasis across the clinical trajectory of breast cancer.

## Discussion

NMR spectroscopy has become firmly established as a robust and reproducible platform for biomarker discovery and quantification in precision medicine, metabolomics, clinical diagnostics, and bioanalytical chemistry.^38^ Although its sensitivity is lower than that of mass spectrometry for detecting metabolites at concentrations below the micromolar range, NMR is widely adopted for the quantification of clinically relevant biomarkers and for monitoring metabolic diseases.^2^ Applications now extend from the detailed characterization of lipoprotein subclasses^6^ and total protein content in the blood^3^ to the quantification of a broad spectrum of biochemically important small-molecule metabolites.^27^ Collectively, these advances, combined with the unique nature of NMR (i.e., high reproducibility and direct quantification of metabolites), have secured NMR as both a clinical tool and a cornerstone of the omics sciences.

In this work, we extend the analytical scope of NMR by introducing an additional dimension: the automated quantification of essential metal ions from plasma- and serum-EDTA samples, thereby providing a panel of clinically important measurements that inform on systemic metal ion homeostasis. While the use of EDTA complexes to measure calcium and magnesium in blood has been previously suggested, here we provide the first comprehensive, multicenter-validated, and fully automated cheminformatic workflow for their robust quantification directly from 1D ^1^H NMR spectra. Beyond Ca^2+^ and Mg^2+^, our algorithm uniquely incorporates trace metal ions, including Zn^2+^, which are increasingly recognized as key biomarkers in a variety of pathological states.^36,39,40^ The method is fully automated, enabling both the identification and deconvolution of metal–EDTA complexes and the removal of interfering EDTA signals, thereby producing metabolomic-ready profiles without manual intervention.

Importantly, the platform is designed with flexible functionality: it can selectively retain metal–EDTA signals when quantification is desired or remove them—together with free EDTA signals—when untargeted metabolomics analysis focusing on the ^1^H NMR profiles of the remaining metabolites is the primary objective. This dual capability is critical because, while EDTA signals provide quantitative insights into ion concentrations, their presence may interfere with metabolites resonating in close spectral proximity. By reconstructing the affected spectral regions, our algorithm systematically reduces artifacts and preserves surrounding spectral integrity. These QC functionalities are central to the platform’s robustness. Automated signal removal and reconstruction minimize metabolic information loss while producing metabolomics-ready spectra that can be directly integrated into high-throughput -omics pipelines without requiring manual preprocessing. Furthermore, consistent handling of EDTA-associated signals ensures harmonization across data sets acquired under different magnetic field strengths, thereby enhancing multicohort comparability.^41^ More importantly, the algorithm can be retrospectively applied to existing data sets containing EDTA in their ^1^H NMR profiles, enabling the re-examination and enhanced utilization of legacy data. This capability provides a unique opportunity to improve QC prior to multivariate analyses and to incorporate metal ion quantification into both targeted and untargeted statistical workflows, thereby extending biomarker discovery to include metallomic components. Together, these features establish a standardized and scalable framework for the integration of metal ion quantification into routine NMR-based metabolomics.

The translational value of this platform was demonstrated through its application to the BSMS breast cancer cohort (∼1000 plasma-EDTA samples), where the algorithm successfully processed all spectra in a high-throughput, quality-controlled manner. This large-scale analysis revealed a significant decline in circulating zinc levels in patients with invasive breast cancer, while calcium and magnesium levels remained unchanged across disease stages. Zinc concentrations did not differ between cancer-free individuals and those with benign tumors, but a marked reduction was observed in invasive cases, consistent with a stage-dependent disruption of zinc homeostasis. Strikingly, these findings are in full agreement with meta-analyses of independent breast cancer cohorts and with recently published studies highlighting zinc dysregulation as both a biomarker and a mechanistic driver of breast cancer progression.^36^

## Conclusions

In summary, our work establishes an NMR-based, fully automated, and multicenter-validated approach for quantifying important metal ions in blood samples containing EDTA. By bridging metabolomics with metal ion homeostasis, the method expands the scope of NMR profiling beyond conventional metabolites and introduces clinically actionable measurements directly into the workflow. Our approach offers a generalizable strategy for investigating the role of metal ion dysregulation across a broad spectrum of diseases (here applied in a large breast cancer cohort), with immediate applications in biomarker discovery, clinical diagnostics, and systems-level omics research.

The software, incorporating all of the above-described features in a user-friendly graphical user interface (GUI) (Figures S13, S14 and Table S4), is freely available to clinicians, chemists, and the metabolomics community at the Github repository and can be downloaded following the link: https://github.com/pantakis/Blood_Metal_Ions_Quant. The software can be retrospectively applied to legacy data sets of EDTA-containing ^1^H NMR profiles. Example data sets, ^1^H NMR serum/plasma-EDTA spectra, and clinical data included in our study, and a reference EDTA spectrum can be found at: 10.6084/m9.figshare.30868295.

## Supplementary Material







## Notes

Concentration ranges of major metal ions in blood, based on normal and abnormal values reported in the Human Metabolome Database (HMDB) (Figure S1); calibration curve for the Zn based upon Atomic Absorption spectrometry (AAS) measurements (Figure S2); Consort diagram of the cohorts used for EDTA-PLATFORM chemical shift models creation (Figure S3); consort diagram of the cohorts used for the automated absolute and relative quantification of metal ions (Figure S4); chemical shifts (δ) values range for EDTA, its complexes and glucose (Figure S5); chemical shifts (δ) assignment strategy (Figure S6); summary statistics of selected chemical shifts prediction models (Table S1); examples of metal ions-EDTA deconvolution (Figure S7); correlation of integrals with metal ions concentrations (Figure S8); metal ions standard curves (Figure S9); validation of absolute metal ions quantification based upon non-normalized integrals (Figure S10); statistical analysis of ions quantification vs independent analyses of multicentered cohorts (Table S2); evaluation of metal ion automated quantification (Figure S11, Table S3); metal ions correlation to age and BMI in BSMS cohort (Figure S12); analysis of the algorithm construction – computational and functional details (Figure S13, Table S4); and graphical user interface – metal ions quantification software (Figure S14) (PDF)
Chemical shifts models training data set (Data set S1) (XLSX)
Chemical shifts models validation data set (Data set S2) (XLSX)
