# Supplementary material for: A Universal Framework for Blood Ionome Extraction and Intelligent Quality Control in 1H NMR Metabolomics
Source: Anal Chem. 2026 Apr 21;98(17):12647–59. doi: 10.1021/acs.analchem.5c08255 (PMC13150807; doi:10.1021/acs.analchem.5c08255)
Supplement: Supplementary file 1 [file ac5c08255_si_001.pdf]

SUPPORTING INFORMATION

A universal framework for blood ionome extraction and intelligent quality control in ^{1}H NMR metabolomics

Anastasios Theodorou,[1,2] Ivan Vučković,[3] Eirini Papadimitriou,[1] Konstantinos Papakonstantinou,[4] Vasiliki Taki,[4] Alexandra Louka,[5] Costas Papaloukas,[2] Constantine D. Stalikas,[1] Nikolaos Giormezis,[4] Fotini Paliogianni,[4] Ian R. Lanza,[3,6] Justin Stebbing,[7,8] and Panteleimon G. Takis[1,9]*

[1]Section of Analytical and Inorganic Chemistry, Department of Chemistry, University of Ioannina, Ioannina 45110, Greece

[2]Department of Biological Applications and Technology, University of Ioannina, Ioannina 45110, Greece

[3] Metabolomics Core, Mayo Clinic, Rochester, Minnesota 55905, United States

[4]School of Medicine and University Hospital, University of Patras, 26504 Rio, Greece

[5]Department of Clinical and Experimental Epilepsy, Queen Square Institute of Neurology, University College London, London, WC1N 3BG, UK

[6]Division of Endocrinology and Metabolism, Mayo Clinic College of Medicine, Rochester, Minnesota 55905, United States

[7]School of Life Sciences, Anglia Ruskin University, East Road, Cambridge CB1 1PT, UK

[8]Department of Surgery and Cancer, Imperial College London, Hammersmith Hospital, Du Cane Road, London W12 0NN, UK

[9]Section of Bioanalytical Chemistry, Division of Systems Medicine, Department of Metabolism, Digestion, and Reproduction, Imperial College London, Hammersmith Hospital, Du Cane Road, London W12 0NN, U.K.

*Correspondence should be addressed at Prof. P. G. Takis: ptakis@uoi.gr or p.takis@imperial.ac.uk

Contents

Table of Contents

Pages

Concentration ranges for different metal ions in blood *S3*

Figure S1 .... S3

Atomic absorption spectrometry calibration curve of zinc *S4*

Figure S2 .... S4

Consort diagram of the cohorts used for chemical shifts models creation *S5*

Figure S3 .... S5

Consort diagram of the workflow for the quantification of the metal ions *S6*

Figure S4 .... S6

Chemical shifts (δ) values range for EDTA, its complexes and glucose *S7*

Figure S5 .... S7

Chemical shifts (δ) assignment strategy *S8*

Figure S6. .... S8

Examples of metal ions-EDTA deconvolution *S9*

Figure S7. .... S9

Correlation of metal ions EDTA NMR deconvoluted integrals (in a.u.) with metal ions absolute concentrations measured by independent techniques *S10*

Figure S8. .... S10

Metal ions standard curves for the 500MHz (non-normalised integrals) *S11*

Figure S9. .... S11

Validation of absolute metal ions quantification based upon non-normalised integrals (at 500 MHz) *S12*

Figure S10. .... S12

Statistical analysis of ions quantification vs independent analyses of multi-centred cohorts *S13*

Table S2 .... S13

Performance evaluation of metal ion automated quantification via our algorithm *S14*

Figure S11. .... S14

Table S3 .... S15

Evaluation of metal ions correlation to age and BMI in BSMS cohort *S16*

Figure S12. .... S16

Analysis of the algorithm construction – computational and functional details *S17*

Figure S13 .... S17

Table S4 .... S18

Python Based Code S19

Metabolite Patterns S19

MATLAB Based code S20

Spectral data preparation S20

Baseline calculation S21

Fitting the metal-EDTA complexes ^{1}H NMR signals S22

Integral Calculation and Metabolite Removal S23

Final Outputs S24

Graphical user interface – metal ions quantification software *S26*

Figure S14 .... S26

References *S27*

Concentration ranges for different metal ions in blood

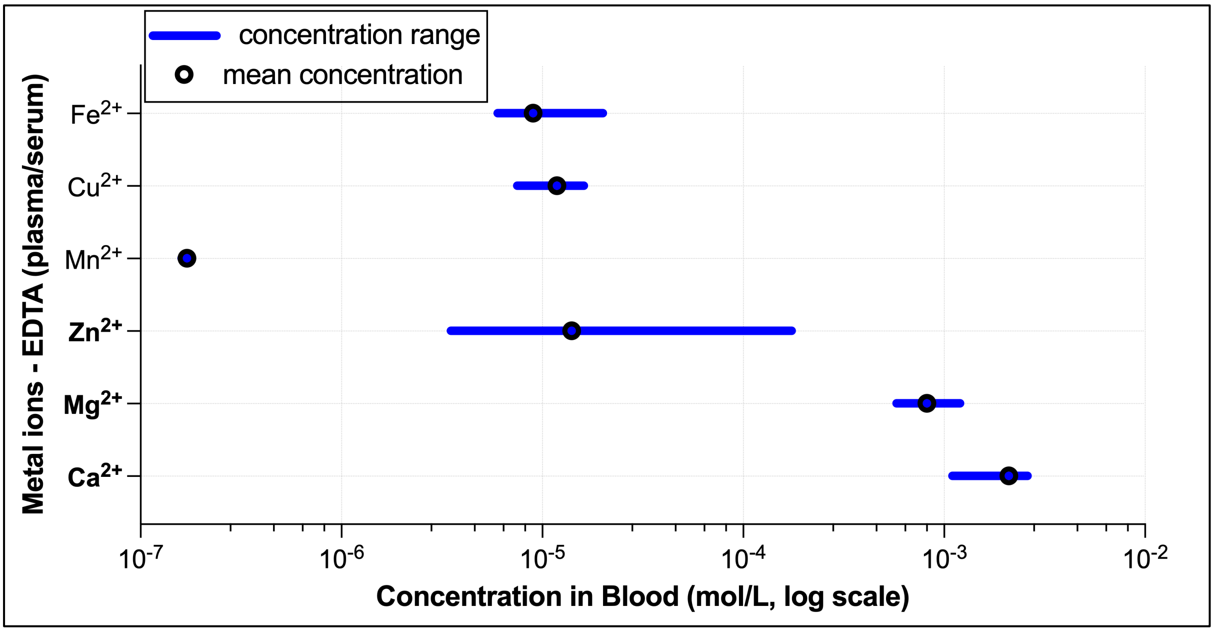

Figure S1. Concentration ranges of major metal ions in blood, based on normal and abnormal values reported in the Human Metabolome Database (HMDB).[1,2] Calcium (Ca^{2+}), magnesium (Mg^{2+}), and zinc (Zn^{2+}) occur at concentrations within the detectable range of ^{1}H NMR spectroscopy (i.e., > ~10 µM), whereas copper, iron and manganese are less abundant to be detected via their chelation with EDTA.

Atomic absorption spectrometry calibration curve of zinc

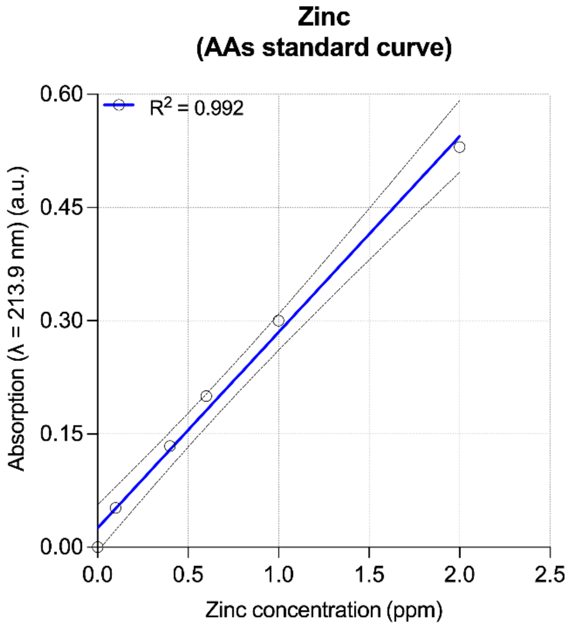

Figure S2. Calibration curve for the Zn based upon Atomic Absorption spectrometry (AAs) measurements. The absorption at 213.9 nm increases linearly with zinc concentration (R^2 = 0.992), confirming the suitability of this signal for the quantitative determination of Zn.

Consort diagram of the cohorts used for chemical shifts models creation

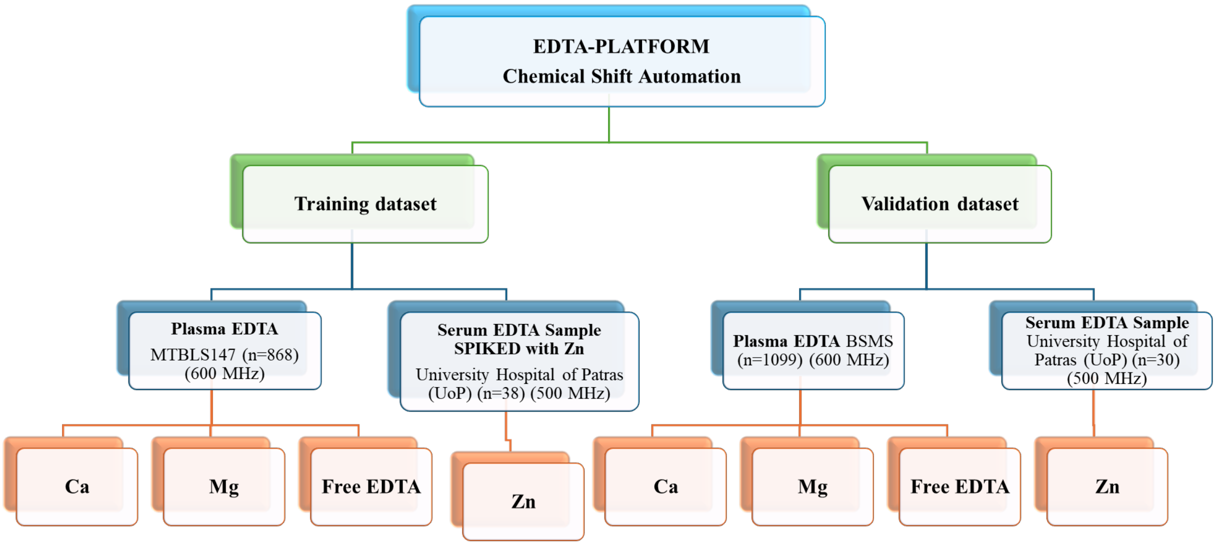

Figure S3. Consort diagram of the cohorts used for EDTA-PLATFORM chemical shift models creation. The training dataset comprised of plasma EDTA samples from the MTBLS147 cohort (n = 868, 600 MHz) and Zn-spiked serum EDTA samples from the University Hospital of Patras (UoP) (n = 38, 500 MHz). The validation dataset included independent plasma EDTA samples from the BSMS cohort (n = 1099, 600 MHz) and serum EDTA samples from UoP (n = 30, 500 MHz). Across both datasets, signals corresponding to Ca-, Mg-, Zn-EDTA complexes and free EDTA were analyzed.

Consort diagram of the workflow for the quantification of the metal ions

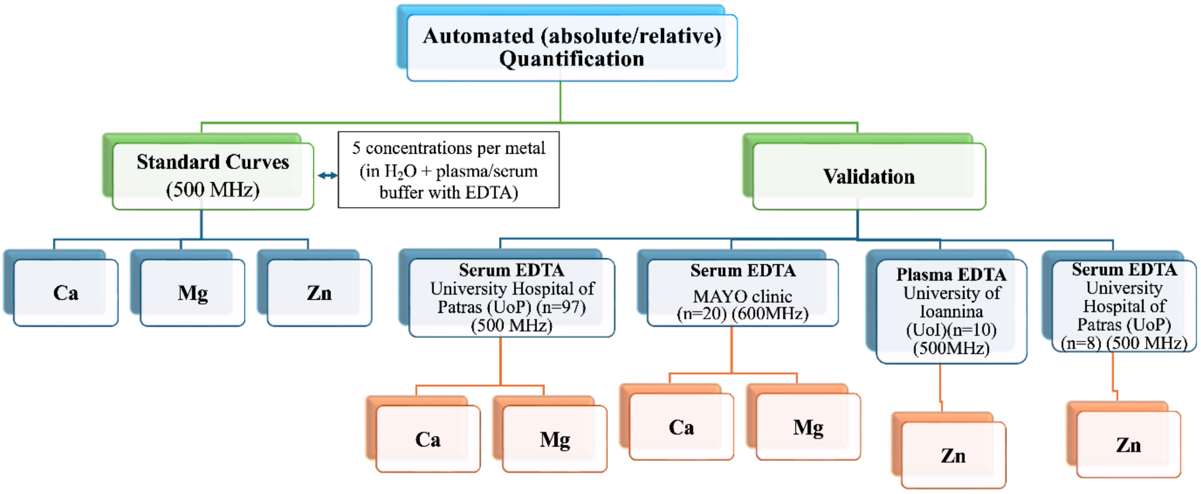

Figure S4. Consort diagram of the cohorts used for the automated absolute and relative quantification of metal ions. Standard curves were generated at 500 MHz for Ca, Mg, and Zn. Validation was performed on independent serum EDTA samples from the Mayo Clinic (n = 20, 600 MHz), plasma EDTA samples from the University of Ioannina (n = 10, 600 MHz), and serum EDTA samples from the University Hospital of Patras (UoP) (n = 10, 500 MHz).

Chemical shifts (*δ*) values range for EDTA, its complexes and glucose

The ^{1}H NMR signals corresponding to free EDTA and its various metal ion complexes were assigned in 868 plasma-EDTA spectra previously recorded and available through the MetaboLights repository[3] (accession number MTBLS147) and 38 internally acquired serum-EDTA spectra (spiked with Zn^{2+} for accurate assignment of Zn-EDTA signals). All NMR samples were prepared following standard operating procedures consistent with those applied in this study.[4] As shown in **Figure S5**, the distribution of chemical shift (*δ*) values extends over approximately 0.02 to 0.1 ppm (depending on the EDTA form), reflecting substantial intrinsic variability within the dataset. This variation, arising from differences in sample composition (e.g., pH, metabolite concentrations, and protein content), ensures that our predictive models were trained on a chemically diverse and representative dataset, thereby enhancing their generalizability and robustness.

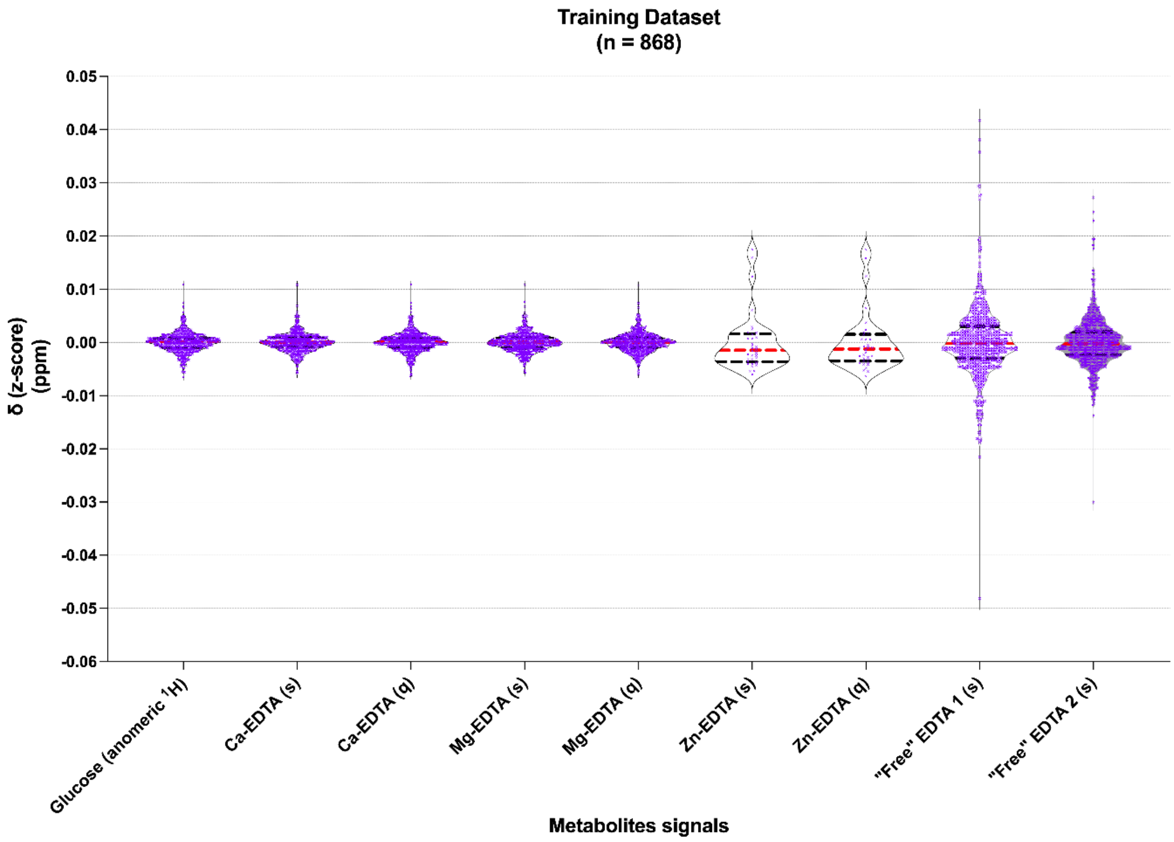

Figure S5. Distribution plots of the chemical shifts (δ) of several spin systems from the plasma-EDTA cohort that was employed for the construction of the δ-prediction models. For data harmonization, the mean chemical shift value of each spin system was subtracted prior to plotting (i.e., z-scored δ values).

Chemical shifts (δ) assignment strategy

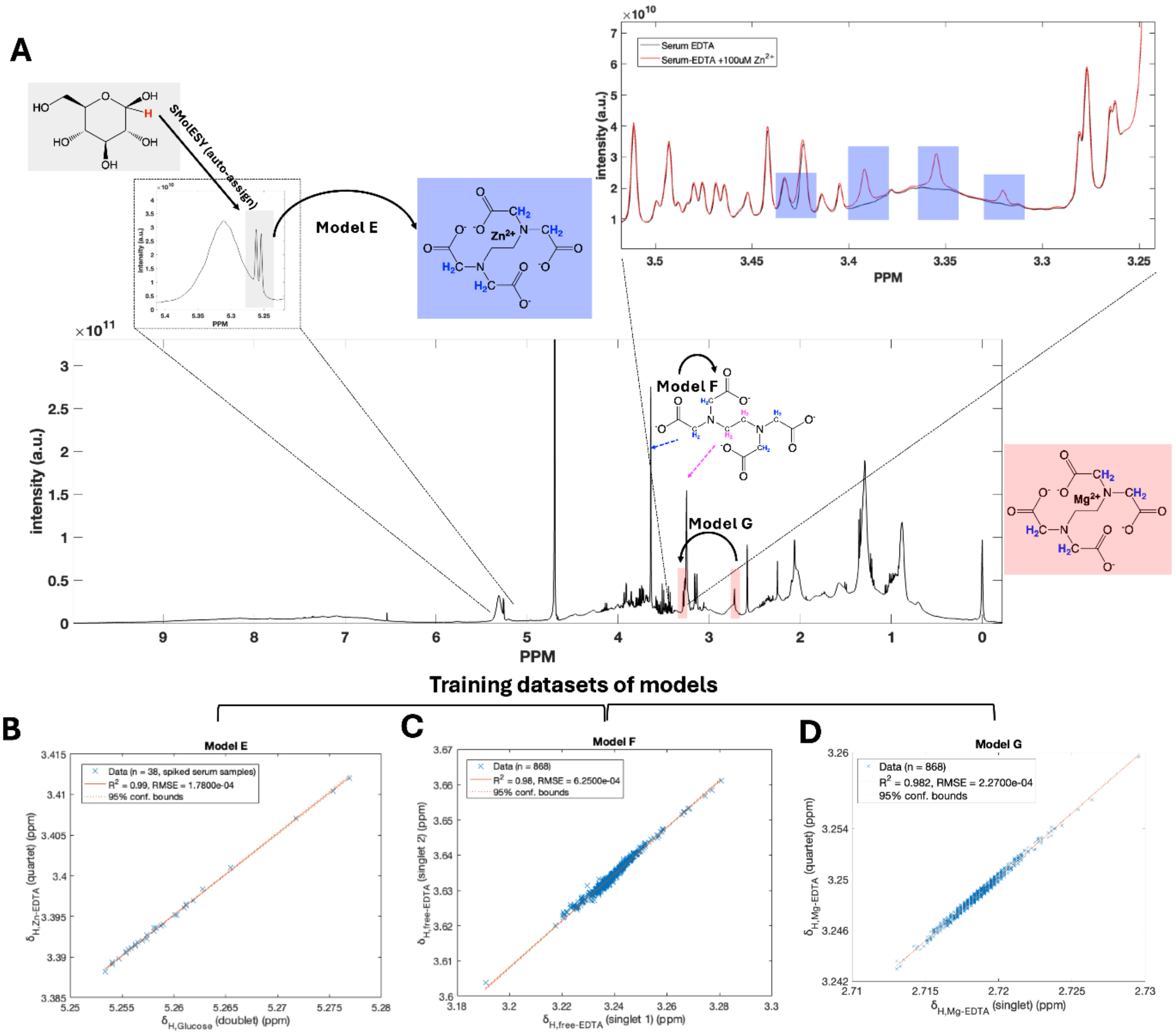

Figure S6. The (**A**) The δ values of the anomeric ^{1}H of glucose (red font) were linearly correlated with (**B**) the quartet δ values of Zn–EDTA protons (blue font) (R^2 = 0.99; model E). (**C**) For free EDTA, both spin systems exhibited near-perfect linear correlations between them (R^2 = 0.98, model F), allowing their assignment when one is auto-assigned as previously indicated.[5] (**D**) the quartet δ values of Mg–EDTA protons (blue font) (R^2 = 0.98; model G). The summary of the model E, F and G statistics are summarised in **Table S1** (see below).

Table S1. Summary statistics of selected chemical shifts (δ) prediction models.

Fitted Model	Adjusted R^2	MSE	RMSE	F-statistic vs. constant model	p-value
Model A	0.982	5.4799e-08	0.000234	4.83e+04	<1.00e-99
Model B	0.967	9.6416e-08	0.000311	2.53e+04	<1.00e-99
Model C	0.996	1.1353e-08	0.000107	2.15e+05	<1.00e-99
Model D	0.999	1.5433e-08	0.000124	6.63e+04	1.21e-54
Model E	0.999	3.1658e-08	0.000178	3.25e+04	1.06e-49
Model F	0.989	6.6942e-07	0.000818	8.32e+04	<1.00e-99
Model G	0.982	5.1473e-08	0.000227	4.82e+04	<1.00e-99

Examples of metal ions-EDTA deconvolution

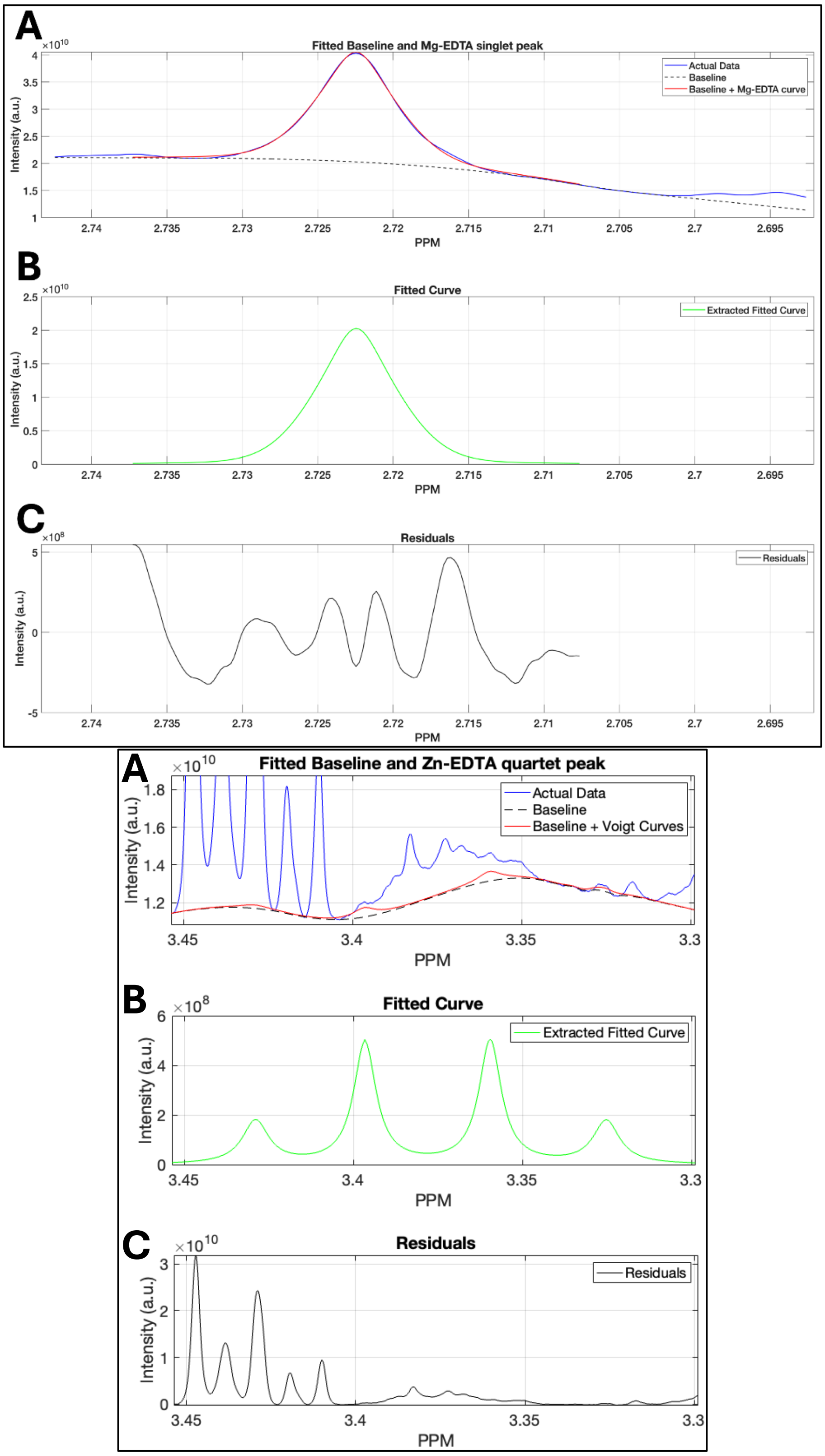

Figure S7. Examples of deconvoluted signals from metal-ions EDTA complexes: (upper panel **A-C**) the case of Mg-EDTA singlet and (bottom panel **A-C**) the case of Zn-EDTA quartet.

Correlation of metal ions EDTA NMR deconvoluted integrals (in a.u.) with metal ions absolute concentrations measured by independent techniques

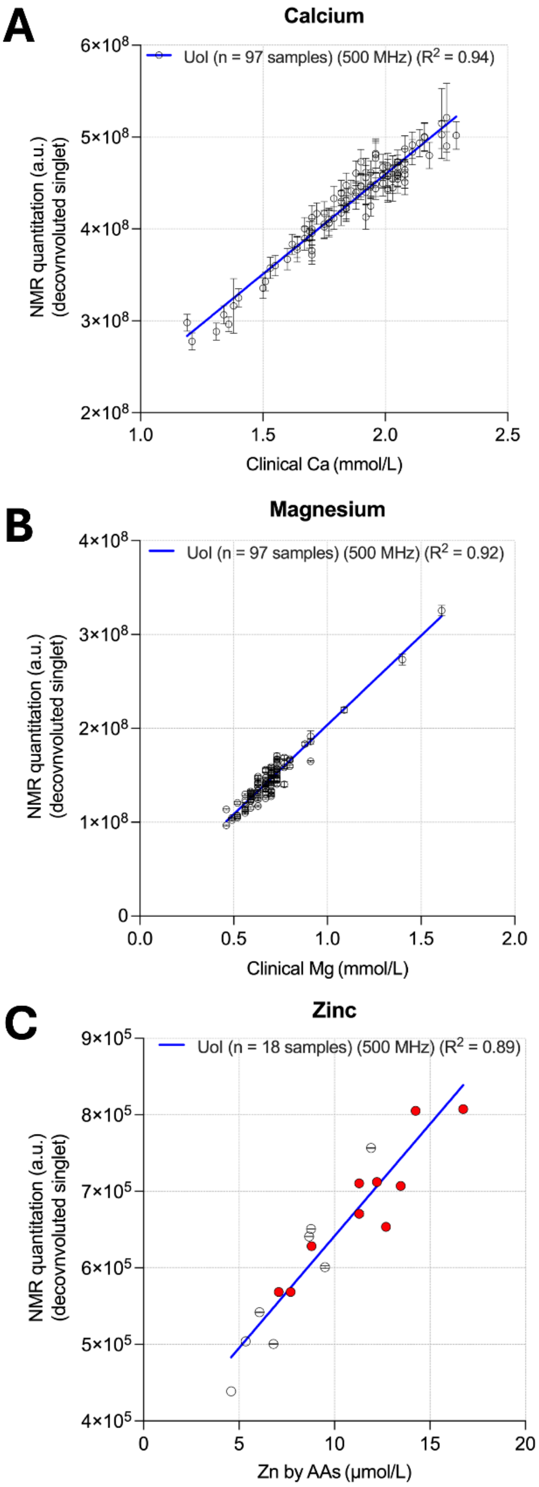

Figure S8. The deconvoluted integrals of specific spin systems from (**A**) Ca-EDTA, (**B**) Mg-EDTA and (C) Zn-EDTA complexes versus clinical/spectroscopic measurements of the corresponding ions in 97 serum samples for Ca^{2+} and Mg^{2+}, and in 18 samples [8 serum (open circles) and 10 plasma-EDTA (red circles) samples] for Zn^{2+}. Results clearly demonstrate that NMR integrals are highly correlated with the independent measurements of metal ions.

Metal ions standard curves for the 500MHz (non-normalised integrals)

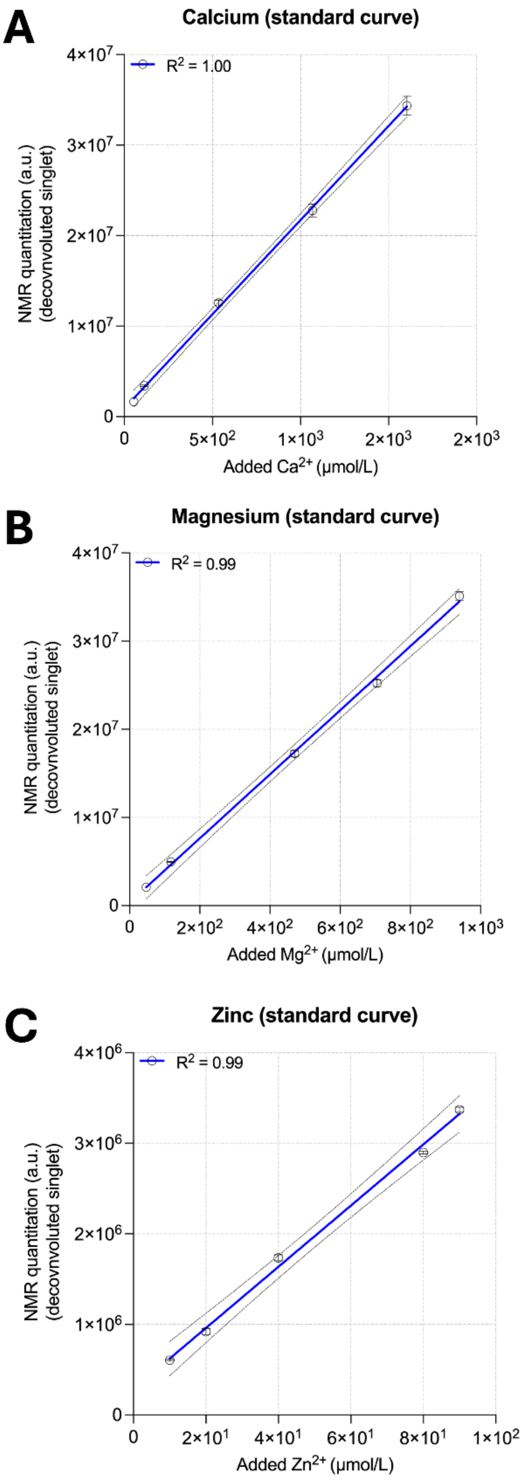

Figure S9. The standard (calibration) curves for: (**A**) calcium, (**B**) magnesium and (**C**) zinc. The curves were built from the fitted (deconvoluted) integrals of one ^1H NMR spin system from each metal ion EDTA complex (i.e., the ^1H NMR singlet) of each in 5 concentrations for each ion in pure, de-ionised water solution, prepared with 50% serum/plasma buffer commonly used for ^1H NMR metabolomics and acquired at 500 MHz ^1H Larmor frequency spectrometer. The concentration range was determined based upon the normal/abnormal concentrations of each metal ion in blood as reported in HMDB.

Validation of absolute metal ions quantification based upon non-normalised integrals (at 500 MHz)

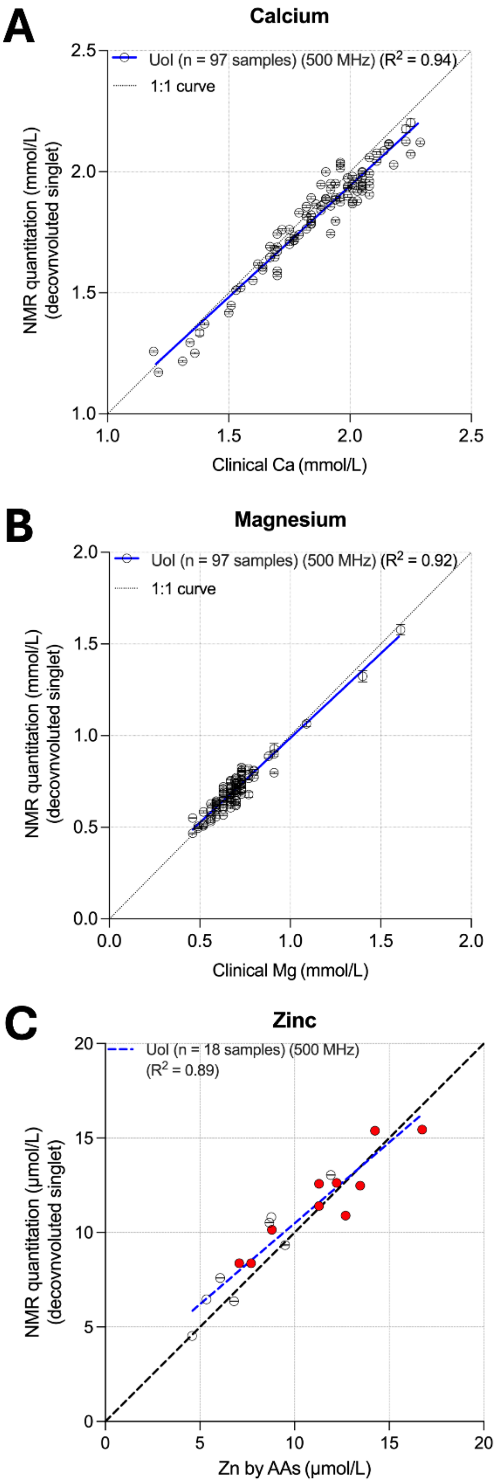

Figure S10. Translation of deconvoluted ^{1}H NMR integrals from (**A**) Ca-EDTA, (**B**) Mg-EDTA and (**C**) Zn-EDTA complexes into absolute concentration values (in mmol/L) via the standard curves that are described in **Fig. S11**. Results are from recorded spectra at 500 MHz. For all cases the clinical/spectroscopic measurements show strong linear correlations with the NMR results (R^2 > 0.90) and align closely with expected 1:1 (diagonal) curve..

Statistical analysis of ions quantification vs independent analyses of multi-centred cohorts

Validation of the automated algorithm for Ca^{2+} and Mg^{2+} quantification was performed using two multicentre cohorts comprising 117 serum-EDTA spectra—97 acquired at 500 MHz (UoI cohort) and 20 at 600 MHz (Mayo Clinic cohort) (see Fig. 4A–B, bottom panels). The results demonstrated strong linear agreement between NMR-derived and clinically measured concentrations across both cohorts, with excellent correlations and near-identical regression lines (see below the **Table S2**), confirming the robustness and reproducibility of the method across instruments and study sites.

Table S2. Testing linear regression coincidence for UoI and MAYO cohorts (ANOVA F-tests) for metal ions absolute quantification in different magnetic fields.

**Testing linear regression coincidence for UoI and MAYO cohorts (ANOVA F-tests)**	
The case of Ca^{2+} absolute quantification by NMR	
Slope	Intercept
F = 3.671, DFn = 1, DFd = 113<br>P=0.0579	F = 0.8804, DFn = 1, DFd = 114<br>P=0.3501
→ If the overall slopes were identical, there is a 5.788% probability of randomly selecting data points that would produce slope differences of this magnitude. Therefore, the observed differences in slopes are not quite statistically significant.<br><br>→ Since the slopes are not significantly different, it is possible to calculate one slope for all the data.<br>The pooled slope equals 0.9148.	→ If the overall elevations were identical, there is a 35.01% probability of randomly selecting data points that would produce slope differences of this magnitude. Therefore, the observed differences in slopes are not statistically significant.<br><br>→ Since the Y intercepts are not significantly different, it is possible to calculate one Y intercept for all the data.<br>The pooled intercept equals 0.1159.
The case of Mg^{2+} absolute quantification by NMR	
Slope	Intercept
F = 0.02365, DFn = 1, DFd = 113<br>P=0.8780	F = 4.62, DFn = 1, DFd = 114<br>P = 0.0701
→ If the overall slopes were identical, there is an 87.8% probability of randomly selecting data points that would produce slope differences of this magnitude. Therefore, the observed differences in slopes are not statistically significant.<br><br>→ Since the slopes are not significantly different, it is possible to calculate one slope for all the data.<br>The pooled slope equals 0.9692.	→ If the overall elevations were identical, there is a 7.01% probability of randomly selecting data points that would produce slope differences of this magnitude. Therefore, the observed differences in slopes are not statistically significant.<br><br>→ Since the Y intercepts are not significantly different, it is possible to calculate one Y intercept for all the data.<br>The pooled intercept equals 0.0401.

Performance evaluation of metal ion automated quantification via our algorithm

The limit of detection (LOD) and quantification (LOQ) of ions were calculated via the following formulas[6] based upon the standard curves of the normalized integrals from metal-EDTA complexes:

$$LOD = 3.3 \times {}^{\sigma}/_{S}\,, \qquad \text{eqn (1)}$$

where σ is the standard deviation of the response and S is the slope of the standard curve of each metal ion as depicted and described in Fig. 4A-C of the main manuscript upper panels.
Accordingly, for the calculation of the LOQ values the following formula was applied:

$$LOQ = 10 \times {}^{std(S)}/_{a}\,, \qquad \text{eqn (2)}$$

where $std(S)$ is the standard deviation of the response, S, and a is the slope of the standard curve of each metal ion as depicted and described in Fig. 4A-C of the main manuscript upper panels.

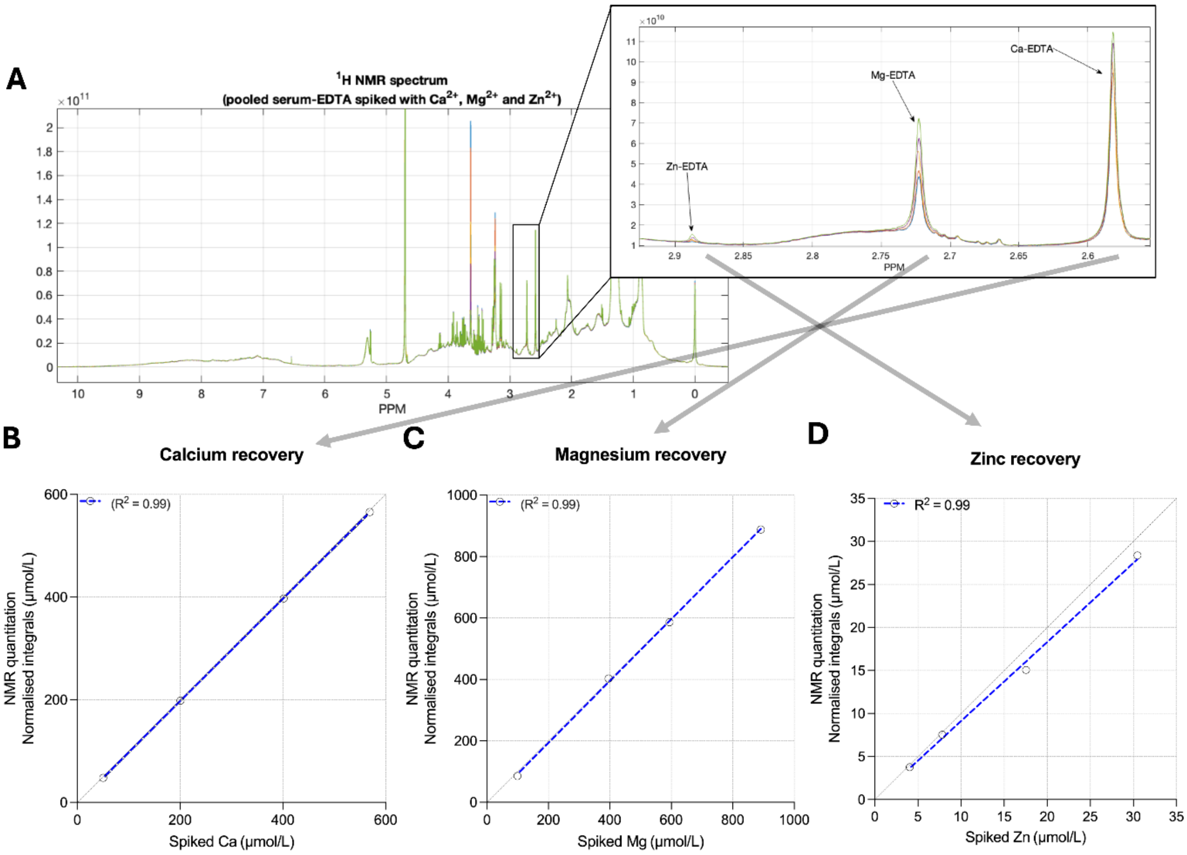

Figure S11. Four different spiked concentrations of all metal ions in a pooled serum-EDTA. (**A**) The acquired ^{1}H NMR spectra, with a zoom into the spectra region containing the Ca-, Mg- and Zn-EDTA characteristic singlets. The linear regression plots of the spiked concentration vs the measured concentration by our algorithm for (**B**) Ca^{2+}, (**C**) Mg^{2+} and (**D**) Zn^{2} clearly demonstrate the high recovery accuracy of our method (see below Table S3).

In addition of the LOD and LOQ values that percent (%) recovery of each metal ion was calculated based upon the simultaneous spiking of the metal ions in *four* different concentrations in a pooled sample of 97 serum-EDTA samples (**Fig. S11A-D**). For each ion, the mean % recovery was obtained by averaging the measured % recovered concentrations produced by our algorithm, following the formula:

$$\%\,Recovery = \frac{Calculated\ metal\ ion\ concentration\ by\ NMR}{Spiked\ metal\ ion\ concentration} \times 100\,, \qquad \text{eqn (3)}$$

The calculated LOD, LOQ and mean percent (%) recovery values are reported in the **Table S3** (see below).

Table S3. Summary of performance characteristics for the metal ions absolute quantification via ^{1}H NMR.

	LOD (µM)	LOQ (µM)	Average Recovery (%)
Calcium	47.25 ± 2.07	143.14 ± 7.94	98.20
Magnesium	39.97 ± 1.69	121.19 ± 7.03	96.73
Zinc	3.40 ± 0.75	10.25 ± 2.38	92.17

Evaluation of metal ions correlation to age and BMI in BSMS cohort

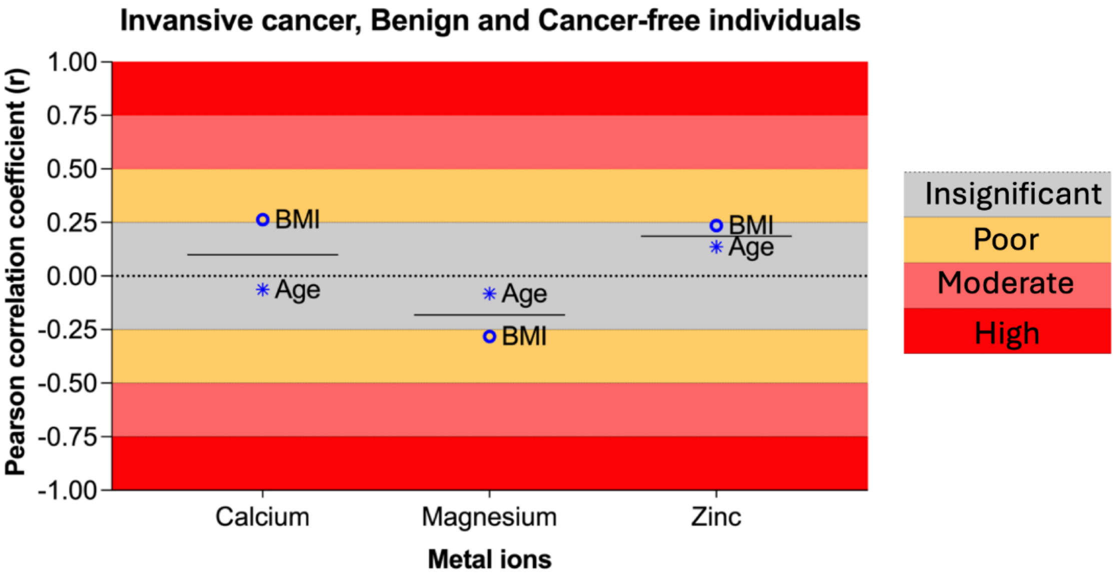

Figure S12. The Pearson correlation coefficients (r) were calculated for the measured concentration of calcium, magnesium and zinc in the plasma-EDTA samples versus the age and BMI of 934 individuals from the BSMS (breast cancer) study.[7] None of the metal ions exhibited statistically significant correlations for both age and BMI of the participants, since all r values were < 0.26.

Analysis of the algorithm construction – computational and functional details

The developed code provides an integrated and automated computational framework for the modeling, fitting, and quantitative analysis of ^1H nuclear magnetic resonance (NMR) spectra focused specifically on free ethylenediaminetetraacetic acid (EDTA) and its metal ion complexes, namely, Ca-EDTA, Mg-EDTA, and Zn-EDTA. The overarching purpose of the framework is to establish a standardized and reproducible pipeline capable of identifying, quantifying, and, when necessary, removing these signals from biological spectra such as human blood serum and plasma.

By maintaining a consistent analytical structure for all EDTA related compounds, the workflow enables systematic spectral interpretation and quantitative comparison across samples and ions, while minimizing user intervention and experimental bias.

As seen in **Fig. S13** and **Table S4**, the process operates in two major stages that are technically distinct yet functionally complementary, combining both Python and MATLAB programming suites. The first stage, implemented in Python, is responsible for the generation of Voigt-based signal models for each compound. These models describe the characteristic spectral

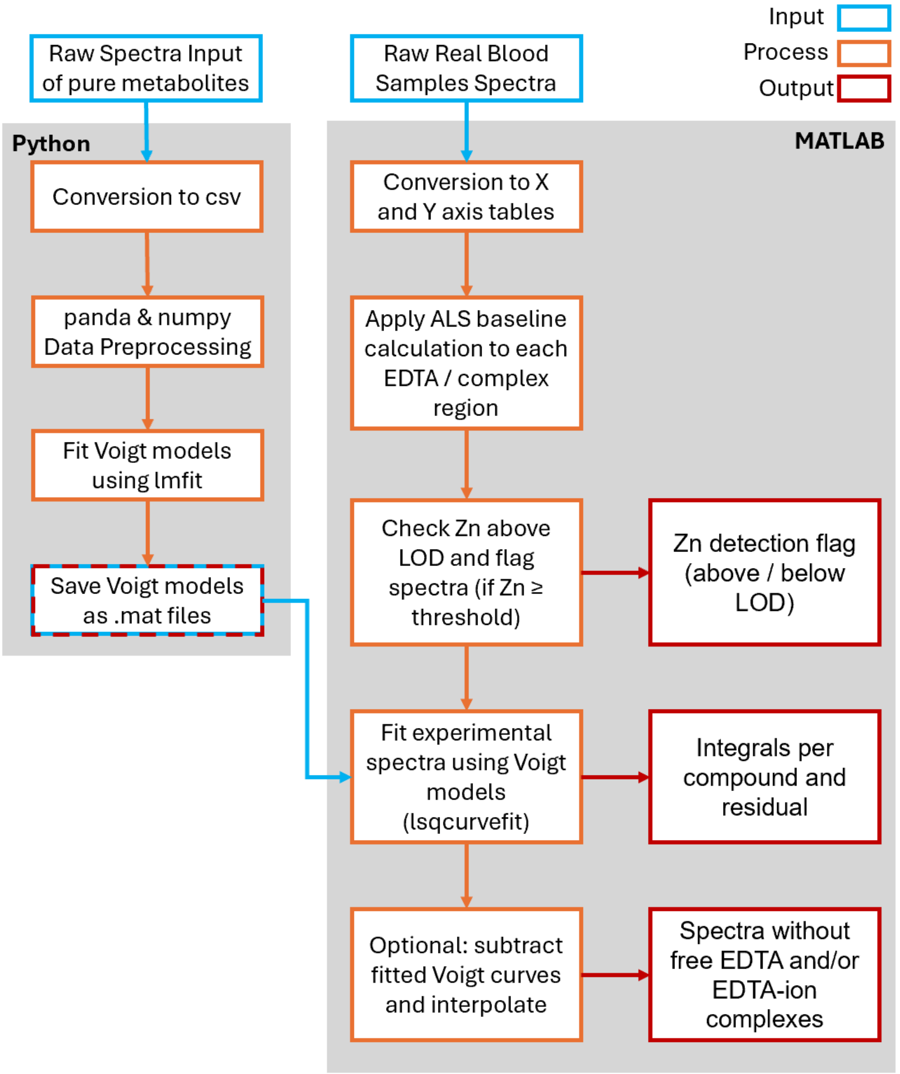

Figure S13. Schematic representation of the full analytical workflow combining Python-based Voigt model generation from pure metabolite spectra with MATLAB-based processing of real blood spectra. The pipeline includes spectral conversion, region-specific ALS baseline correction, Zn detection, Voigt curve fitting, quantitative integral extraction, and optional subtraction of EDTA and EDTA–ion complex resonances.

Table S4. Summarized python and MATLAB components used for the deconvolution processes

Python Packages	MATLAB Functions and Toolboxes
**pandas**	lsqcurvefit (from Optimization Toolbox)
**numpy**	pchip, interp1, and smoothdata (from Curve Fitting and Signal Processing Toolboxes)
**os**	findpeaks and numerical functions (diff, mean, etc.) (from Signal Processing and Base MATLAB)
**json**	plot, subplot, legend, and axis (from Graphics and Plotting)
**lmfit**	load, save, matfile, mkdir, isfile (from MATLAB I/O and File Management)
**plotly.graph_objects**	
**scipy.io.savemat**	
**joblib**	

features of free EDTA and its metal complexes in a mathematically standardized form. For each ion complex, the script isolates the relevant region of a high-purity reference spectrum—one that contains only the free EDTA or the specific EDTA ion complexes to ensure that the resulting parameters represent uncontaminated signal profiles.

Using the *pandas* library for structured data handling, numpy[8] and scipy[9] for numerical computation, the spectral data are converted into chemical shift and intensity arrays. To correct baseline distortions typical of NMR data, an asymmetric least squares (ALS) smoothing algorithm[10] is applied, generating a flattened baseline from which true peaks can be fitted accurately.

The signal fitting itself relies on Voigt-profile functions, which are mathematical combinations of Gaussian and Lorentzian line-shape components. This allows simultaneous modelling of homogeneous and inhomogeneous broadening effects. Fitting is performed using the *lmfit* library[11], which optimizes the line-shape parameters, Gaussian width (σ), Lorentzian width (γ), peak center, and amplitude, by minimizing the difference between the experimental and theoretical spectra. The resulting model is composed of one or more Voigt profiles corresponding to the individual resonances of the metal-EDTA complex (singlets or quartets), depending on the coordination state of the metal center.

For each compound, the model parameters are stored in serialized format (.pkl) for direct reuse and in MATLAB-compatible format (.mat) for integration into the second part of the analysis workflow.

The second stage, implemented in MATLAB, automates the application of these pre-fitted Voigt models to real biological spectra. Each spectrum is processed by reading its chemical shift axis and intensity data, predicting the expected regions of the free EDTA and its Ca^{2+}, Mg^{2+}, and Zn^{2+} complexes, and performing local ALS baseline correction. The pre-generated Voigt models from the Python stage are then adapted to the corresponding spectral regions. Each fit produces optimized parameters, integral values (representing quantitative peak areas), and diagnostic plots that summarize fitting quality.

When the optional signal(s) removal mode is enabled, the fitted peaks, representing the EDTA related signals, are subtracted directly from the spectrum, followed by interpolation and smoothing to restore spectral continuity. This enables both quantification and spectral cleaning, ensuring that signals from EDTA and its complexes do not interfere with subsequent metabolite analysis.

A key advantage of this framework lies in its universality and reproducibility. Instead of designing separate scripts for each metal complex, the same computational logic, baseline correction, Voigt fitting, integral calculation, and optional removal, is applied uniformly across all EDTA related

species. Only the initial parameters (chemical shift centers and expected multiplicities) differ between models, while all other algorithmic components remain constant.

This uniform design ensures that the fitting and quantification of Ca-EDTA, Mg-EDTA, and Zn-EDTA complexes are performed under identical mathematical and numerical conditions, making cross-comparison between ions both reliable and statistically meaningful. Furthermore, since all results are saved in structured output formats, the fitted parameters and cleaned spectra can be seamlessly integrated into subsequent MATLAB workflows for visualization, statistical analysis, or machine-learning–based postprocessing.

Through this standardized two-part framework, the modelling and quantification of EDTA and its metal complexes become a consistent, transparent, and automatable process. By combining Python's flexibility in data modelling and MATLAB robustness in numerical analysis and visualization, the system provides a comprehensive and reproducible approach to NMR spectral fitting and correction that can be readily extended to other ligand–metal systems using the same foundational code structure.

Python Based Code

Metabolite Patterns

The workflow is structured around a modular and hierarchical design that ensures every EDTA related model is processed through the same sequential framework, from data import and preprocessing to model fitting, parameter storage, and later application on biological spectra. This base skeleton was developed to be general, meaning that the same computational logic applies equally to free EDTA and its metal ions complexes (Ca-EDTA, Mg-EDTA, and Zn-EDTA). The only differences between them lie in their expected chemical shift regions and in the number of resonance components that define their characteristic spectral patterns.

The process begins with the Python component, which generates the initial Voigt models that describe the resonance structure of each compound. The script starts by importing one-dimensional NMR spectral data, stored as CSV files, that contain the chemical shift values (in parts per million, ppm) along the x-axis and the corresponding signal intensities along the y-axis. The library *pandas* is used to read and manage these tabular datasets efficiently, while *numpy* performs the array handling and numerical calculations required for data conversion and manipulation. The *os* library manages file and directory operations, automatically locating input spectra, creating output folders, and ensuring all resulting files are properly organized. To maintain consistency across runs, the initial parameters that define peak centres, line widths, and amplitude ranges are read from a configuration file using *json*.

Once the data and configuration parameters are loaded, the code isolates the spectral region corresponding to the specific compound being modelled. For instance, the region selected for the Ca-EDTA complex includes the resonance of its AB quartet and singlet peaks, while free EDTA occupies a narrower single-resonance window. The script trims the full spectrum to this window so that all further operations are applied only to the relevant portion.

Before fitting, the baseline of the extracted region is corrected using the ALS algorithm. This method iteratively estimates and subtracts the baseline by minimizing a penalized least-squares function that suppresses positive deviations caused by peaks. The corrected spectrum obtained after this step serves as the input for the Voigt model fitting.

The fitting process is performed using *lmfit* function, which provides a high-level interface for non-linear least-squares optimization. Each resonance is modelled using the Voigt function, a profile that combines both Gaussian and Lorentzian components to represent the two principal types of line broadening observed in NMR signals. These functions are defined as:

$$G(x) = \frac{1}{\sigma\sqrt{2\pi}} \exp\left[-\frac{(x-\mu)^2}{2\sigma^2}\right] \qquad \text{eqn (4)}$$

$$L(x) = \frac{1}{\pi} \frac{\gamma}{(x-\mu)^2+\gamma^2} \qquad \text{eqn (5)}$$

where μ is the peak center, σ is the Gaussian width parameter (reflecting inhomogeneous broadening), and γ is the Lorentzian half-width at half-maximum (HWHM) associated with homogeneous broadening. Since both line shapes contribute to the total signal, their combined full width at half maximum (FWHM) is estimated using the empirical relation by Ida et al.:[12]

$$\text{FWHM}_V \approx 0.5346(2\gamma) + \sqrt{0.2166(2\gamma)^2 + (2.355\sigma)^2} \qquad \text{eqn (6)}$$

This approximation allows the script to express the overall linewidth of a Voigt profile as a function of its Gaussian and Lorentzian components, facilitating comparison of signal widths among free EDTA and its metal ion complexes even when acquired at slightly different resolutions.

To simplify numerical computation, the extended pseudo-Voigt approximation is applied. It expresses the Voigt profile as a weighted sum of Gaussian and Lorentzian contributions, with the mixing coefficient η determined by the ratio of their widths:

$$\eta = 1.36603(\frac{\text{FWHM}_L}{\text{FWHM}_V}) - 0.47719(\frac{\text{FWHM}_L}{\text{FWHM}_V})^2 + 0.11116(\frac{\text{FWHM}_L}{\text{FWHM}_V})^3 \qquad \text{eqn (7)}$$

where $\text{FWHM}_L = 2\gamma$ and FWHM_V is the total Voigt width obtained from the previous equation. Using this factor, the complete line shape used for the fitting is written as:

$$V(x) = A[\eta\, L(x) + (1 - \eta)\, G(x)] \qquad \text{eqn (8)}$$

where A represents the amplitude of the peak. This formula corresponds to the final pseudo-Voigt function implemented in the fitting routine. Each resonance within the selected region is modelled by one such function, and in cases like the Ca-EDTA complex, multiple components (four lines) are summed to form a composite profile.

All parameters are initialized from values optimized on a reference compound, typically acetone, which serves as a calibration standard for instrument resolution. The composite Voigt model is then fitted to the experimental data using *lmfit* optimization routines, adjusting the parameters μ, A, σ, and γ to minimize the residuals between the observed and theoretical spectra. The fitting results are validated through residual analysis and the coefficient of determination (R^2) to confirm model accuracy.

After fitting, *scipy.io.savemat* exports the optimized parameters, fitted curves, and x–y data arrays to MATLAB-compatible .mat files, enabling direct integration with the subsequent MATLAB-based workflow. The complete fitting object is serialized using *joblib* to allow for reloading and re-plotting without refitting, improving computational efficiency. Interactive visualizations of the raw spectrum, fitted curve, and residuals are generated using *plotly.graph_objects*, allowing the user to examine the model's performance within a web browser. The *os* package manages all output file generation and directory handling to maintain a structured organization of results.

Through this workflow, the Python script establishes a universal modelling framework that can be applied to free EDTA and each of its metal ion complexes. By maintaining a consistent sequence of operations, data import, baseline correction, Voigt fitting, result storage, and visualization, the base skeleton ensures methodological uniformity and reproducibility across all modelled species. This design allows new metal ions EDTA complexes or related ligands to be incorporated simply by specifying their chemical shift regions and expected multiplicities, while the computational backbone of the analysis remains unchanged.

MATLAB Based code

Spectral data preparation

Once the Voigt models have been created and saved by the Python script, the MATLAB component begins by applying these models to actual experimental spectra. The function first loads the .mat files produced by the Python stage, which contain the pre-optimized Voigt parameters for each EDTA

related compound. These parameters serve as fixed starting points for fitting the corresponding signals in biological samples, ensuring that all subsequent analysis remains consistent with the calibration performed during model generation.

At initialization, the MATLAB script loads a supporting file containing additional pre-trained components or predictive models, such as regression models that help identify expected peak locations based on internal spectral references. It then retrieves the list of all one-dimensional NMR spectra to be analysed from the user-specified directory using a helper routine that reads the individual sample titles and intensity arrays. To maintain clear organization of results, the function automatically generates output folders where the fitted spectra, cleaned spectra, and numerical results will be stored.

For each input spectrum, the function loads both the x-axis (chemical shift) and y-axis (signal intensity) data, along with any relevant acquisition metadata such as the magnetic field strength. Before performing any correction or fitting, the function first determines internal reference signals used for alignment and prediction. This includes the identification of a reliable resonance, such as the glucose doublet, which acts as a positional reference point for the starting the predicting of the rest of the signals of EDTA. Using this internal standard, the script predicts the expected locations of the free EDTA etc. peaks and the rest of the signals are predicted by the chemical shift prediction models (as described above) within the current spectrum by scaling and shifting the known model centers derived from the Python stage.

This predictive step ensures that the fitting region is automatically located, accounting for the chemical shifting changes from spectrum to spectrum. Once the predicted positions for free EDTA and each metal-EDTA complexes are identified, the function isolates those regions and stores them temporarily for further processing. Only after these preparations—loading, organizing, predicting, and isolating the correct spectral segments—does the MATLAB script proceed to the next stage: the baseline calculation and subsequent Voigt fitting, which are applied individually to each isolated region.

Baseline calculation

After the prediction and isolation of the spectral regions corresponding to free EDTA and its metal ion complexes, the MATLAB function performs baseline correction on each segment to remove low-frequency variations and instrumental drift that can distort the true signal shape. This step is essential, as an uneven baseline can significantly affect the accuracy of Voigt fitting and subsequent integral quantification.

The baseline correction is implemented using the ALS smoothing algorithm. The ALS method provides a computationally efficient way to estimate a smooth baseline even in spectra with overlapping peaks. It works by fitting a smooth function z to the observed signal y, while applying asymmetric weighting so that peaks are treated as positive deviations rather than as part of the baseline.

Mathematically, the baseline z is obtained by minimizing the following objective function:

$$\min_z \sum_i w_i(y_i - z_i)^2 + \lambda \sum_i (\Delta^2 z_i)^2 \qquad \text{eqn (9)}$$

where y_i is the observed signal intensity at each point i, w_i is the weight vector that determines the influence of each point on the baseline, λ is a smoothing parameter that controls the stiffness of the baseline, and Δ^2 is the second-order difference operator that approximates the second derivative of z. The first term enforces closeness between the baseline and the observed data, while the second penalizes curvature, ensuring smoothness.

The asymmetric property of the ALS method arises from the iterative updating of the weight vector w. After each iteration, the weights are adjusted according to the residuals between the observed signal and the current baseline estimate:

$$w_i = \{ \begin{matrix} p, & \text{if } y_i > z_i \\ 1-p, & \text{if } y_i \leq z_i \end{matrix} \qquad \text{eqn (10)}$$

where p is the asymmetry parameter, typically set to a small value ($p \ll 0.5$) to ensure that points above the baseline—corresponding to true spectral peaks—are given less weight. This iterative process continues until convergence, producing a smooth baseline that follows the general curvature of the spectrum without fitting the peaks themselves.

In the context of the EDTA and metal-EDTA complexes, this method is applied locally to each isolated spectral region. For example, the baseline around the Zn-EDTA singlet is corrected separately from that of the Ca-EDTA quartet to account for their distinct peak structures and neighbouring resonances. The parameters λ and p are predefined for each compound to ensure optimal smoothing. Larger λvalues produce flatter baselines for regions with broad signals such as the Ca-EDTA quartet, while smaller λ values allow finer adjustment in narrower regions like the free EDTA singlets.

Once the baseline z is calculated, it is subtracted from the observed spectrum y to yield a corrected signal:

$$y_{\text{corrected}} = y - z \qquad \text{eqn (11)}$$

The resulting flattened spectrum accurately represents only the true resonances of the compound without background distortion. This corrected signal is then passed to the Voigt fitting stage, where the pre-defined models generated in Python are applied.

The advantage of using the ALS baseline correction lies in its flexibility and reproducibility. Because it is purely algorithmic and does not depend on manual anchor points, the same approach can be applied consistently across hundreds of spectra, ensuring that every free EDTA, Ca-EDTA, Mg-EDTA, and Zn-EDTA signal is fitted under identical baseline conditions. The algorithm's asymmetry ensures that only peaks are treated as positive deviations while the underlying noise and drift are modelled as part of the baseline.

This step completes the preprocessing stage of the MATLAB workflow. After the baseline has been subtracted, the function proceeds to fit the Voigt profiles to each corrected region, quantify the integrals, and, if requested, perform spectral subtraction to remove the EDTA related signals from the sample.

Fitting the metal-EDTA complexes ^{1}H NMR signals

Once the baseline-corrected spectra have been prepared, the MATLAB function performs Voigt fitting to reconstruct the spectral features of the free EDTA and its metal ion complexes. The fitting procedure directly applies the models previously created in Python, ensuring that all signals are analysed using the same mathematical representation and parameter structure. This integration maintains complete consistency between model generation and model application, minimizing user input and reducing the risk of subjective bias during spectral interpretation.

At this stage, the MATLAB function loads the *.mat* files containing the Voigt model parameters generated by the Python script. These files include the initial estimates for the amplitude, Gaussian width (σ), Lorentzian width (γ), and peak center (μ) of each resonance. Using these reference parameters as starting values, the code fits the experimental data to the corresponding Voigt profile by minimizing the residual difference between the observed and predicted intensities. The fitting optimization is performed using *lsqcurvefit*, which iteratively refines each parameter until convergence is achieved. The process is bounded within physically meaningful limits: the amplitude must remain positive, the widths can vary only within a limited proportional range, and the peak centers are allowed to shift slightly to accommodate minor frequency drifts between spectra.

Each of the EDTA related compounds is fitted using the same computational logic but with structural differences that reflect their unique NMR features. The free EDTA model is the simplest, consisting of two singlets resonances, which serves as the reference for line-shape stability and for validating the overall pipeline. The Ca-, Mg-, and Zn-EDTA complexes are more complex, displaying

both singlet and quartet signals corresponding to different coordination environments within the metal–ligand complex. In these cases, the MATLAB function first fits the singlet resonance, which provides stable amplitude and width estimates, and then reconstructs the quartet automatically using predefined parameters (i.e., *J*-coupling constants) imported from the Python model. These separations are slightly adjusted to match the sampling resolution of the spectrum being analyzed.

These separations are scaled according to the sampling frequency of the input spectrum to maintain correct spacing when the data resolution differs from that used during model creation, using the relationship

$$\mathrm{d_{out}} = \mathrm{d_{in}} \times \frac{\mathrm{f_{out}}}{\mathrm{f_{in}}} \quad \text{eqn (12)}$$

where d_{in} is the original spacing between peaks, and f_{in} and f_{out} are the input and output sampling frequencies, respectively. This adjustment ensures that the reconstructed multiplets remain consistent with the experimental resolution.

For the Zn-EDTA complex singlet, an additional adjustment is made prior to fitting since it partially overlaps with a part of asparagine's (ASN) signal. To minimize interference, the MATLAB function performs a preliminary subtraction step using a predefined ASN model before applying the Voigt fit to the Zn-EDTA singlet region. This ensures that the singlet of the Zn-EDTA resonance is fitted independently of partially overlapped metabolite peaks.

Once the optimal parameters are found, the MATLAB function evaluates the quality of each fit by comparing the modelled and observed spectra. It calculates the residual error and coefficient of determination (R^2) for diagnostic purposes, ensuring that the fitted signal closely matches the experimental data. For signals that deviate significantly from their expected shape or integral, automatic correction is applied by scaling the line widths (σ and γ) proportionally to match the target area defined by the original Python model. This correction preserves the peak geometry while compensating for minor differences in sample line broadening or instrumental resolution.

The final fitted parameters: amplitude, line widths, and peak center, are stored for each spectrum, along with the reconstructed Voigt curve and residuals. These outputs are written to MATLAB compatible arrays for later analysis, allowing comparison of peak positions, widths, and intensities across different spectra. Fitting plots for each compound are also generated and saved automatically, providing a visual record of each optimized model and its performance.

Overall, the fitting step ensures that every EDTA related compound is processed under identical numerical and methodological conditions. Although each metabolite exhibits distinct resonance structures, the shared Voigt fitting framework provides a uniform analytical approach. This consistency enables reliable quantitative comparison between free EDTA and its metal ion complexes, ensuring that observed differences in intensity or width reflect real chemical or physical variations rather than artifacts of the fitting process.

Integral Calculation and Metabolite Removal

After each signal has been successfully fitted, the MATLAB function proceeds to calculate the area under the fitted Voigt profile, which represents the integral of the peak. This integral is directly proportional to the relative abundance of the compound in the sample and forms the basis for quantitative analysis of free EDTA and its metal ion complexes.

The integration is performed numerically by summing the intensity values of the final fitted curve within the defined chemical shift limits. Since the Voigt model already accounts for both Gaussian and Lorentzian broadening, no additional correction or baseline adjustment is required at this stage. For each compound, the function records the integral together with the corresponding fitted parameters, including the final amplitude, Gaussian width (σ), Lorentzian width (γ), and peak center (μ). These values are exported into structured arrays and saved for further statistical analysis and visualization.

The integrals of the fitted signals are stored separately for each compound and resonance type. For example, in the case of the Ca-EDTA complex, the MATLAB function produces two sets of integrals: one for the singlet and another for the AB quartet components. Similarly, a set of two integrals of each deconvoluted ^{1}H NMR signal (one singlet and one AB quartets) from Mg- and Zn-EDTA complexes as well as a set from free EDTA (2 singlets) are produced. This separation allows for direct comparison between coordination environments and between complexes within the same dataset. Although only singlets are used for the metal ions quantification, the rest of the integrals are used for signals deconvolution test of robustness for our algorithm fitting processes.

Once the integrals have been computed, the script optionally performs a spectral removal step to eliminate the fitted Voigt curves from the original spectrum. This operation is particularly important for quality control or for preparing spectra for subsequent metabolite analysis where EDTA related signals may obscure nearby resonances. When the removal option is enabled through the user interface, the MATLAB function subtracts the fitted Voigt profile from the baseline-corrected spectrum, effectively removing the entire signal associated with the compound.

Before interpolation, the function removes a narrow region around each peak center equal to ±2×the fitted linewidth (FWHM/2). This ensures that residual distortions at the center of the subtracted curve are excluded from the reconstruction. The remaining region is then interpolated using the piecewise cubic Hermite interpolation (PCHIP) method, which preserves local monotonicity and spectral curvature. After interpolation, a secondary asymmetric least squares baseline correction is applied to the interpolated region to restore local smoothness and ensure seamless continuity with the surrounding spectrum.

After subtraction, the function applies local interpolation to reconstruct the spectral region where the peak was removed. The interpolation is performed using the PCHIP method. To further refine the result, the function re-applies a light ALS smoothing pass over the affected region, ensuring that the continuity of the spectral baseline is preserved.

This signal removal step can be applied selectively to each compound. For example, one free EDTA singlet is often subtracted to clean the low-field region of the spectrum, while the AB quartets of the Ca-, Mg-, and Zn-EDTA complexes can be removed when they overlap with other metabolite peaks. The subtraction of the Zn-EDTA complex is particularly useful because its resonance partially overlaps with asparagine, and its removal facilitates clearer quantification of the latter.

Following the subtraction, the function saves both the cleaned and unaltered spectra for comparison. The cleaned version, referred to as the corrected or residual spectrum, is stored as *Y_cleaned*, while the original fitted spectrum is stored as *Yaxis*. Alongside these, the function records the x-axis values (*Xaxis*) and the corresponding list of sample identifiers to maintain traceability across datasets.

This combined procedure of integral calculation and optional Voigt curve removal completes the analytical loop between model-based quantification and spectral preprocessing. It ensures that all EDTA related signals—whether free ligand or metal complex—can be quantified accurately and, if required, subtracted in a controlled and reproducible manner. By applying the same operations to every compound and sample, the workflow guarantees that any observed spectral differences arise from real biochemical variations rather than inconsistencies in processing or model implementation.

Final Outputs

At the conclusion of the workflow, the complete set of results is systematically stored and organized for downstream analysis and record keeping. For each processed spectrum, the MATLAB function outputs the original baseline-corrected data (Yaxis), the cleaned spectrum after Voigt removal (Y_cleaned), and the corresponding chemical shift axis (Xaxis). The fitted parameters for each resonance—amplitude, Gaussian and Lorentzian widths (σ and γ), and peak center (μ)—are exported together with the calculated integrals for the free EDTA, Ca-EDTA, Mg-EDTA, and Zn-EDTA complexes. Diagnostic plots showing the fitted and residual spectra are generated for visual verification, and a binary flag identifies spectra in which Zn was detected above the noise threshold. All numerical results are saved in MATLAB compatible .mat files to ensure interoperability with other analytical pipelines,

while the original model structures remain stored in .pkl format for reproducibility. This organized output set provides both quantitative and graphical documentation of every fitting and subtraction event, enabling consistent evaluation, comparison, and integration into broader metabolomic analyses.

Graphical user interface – metal ions quantification software

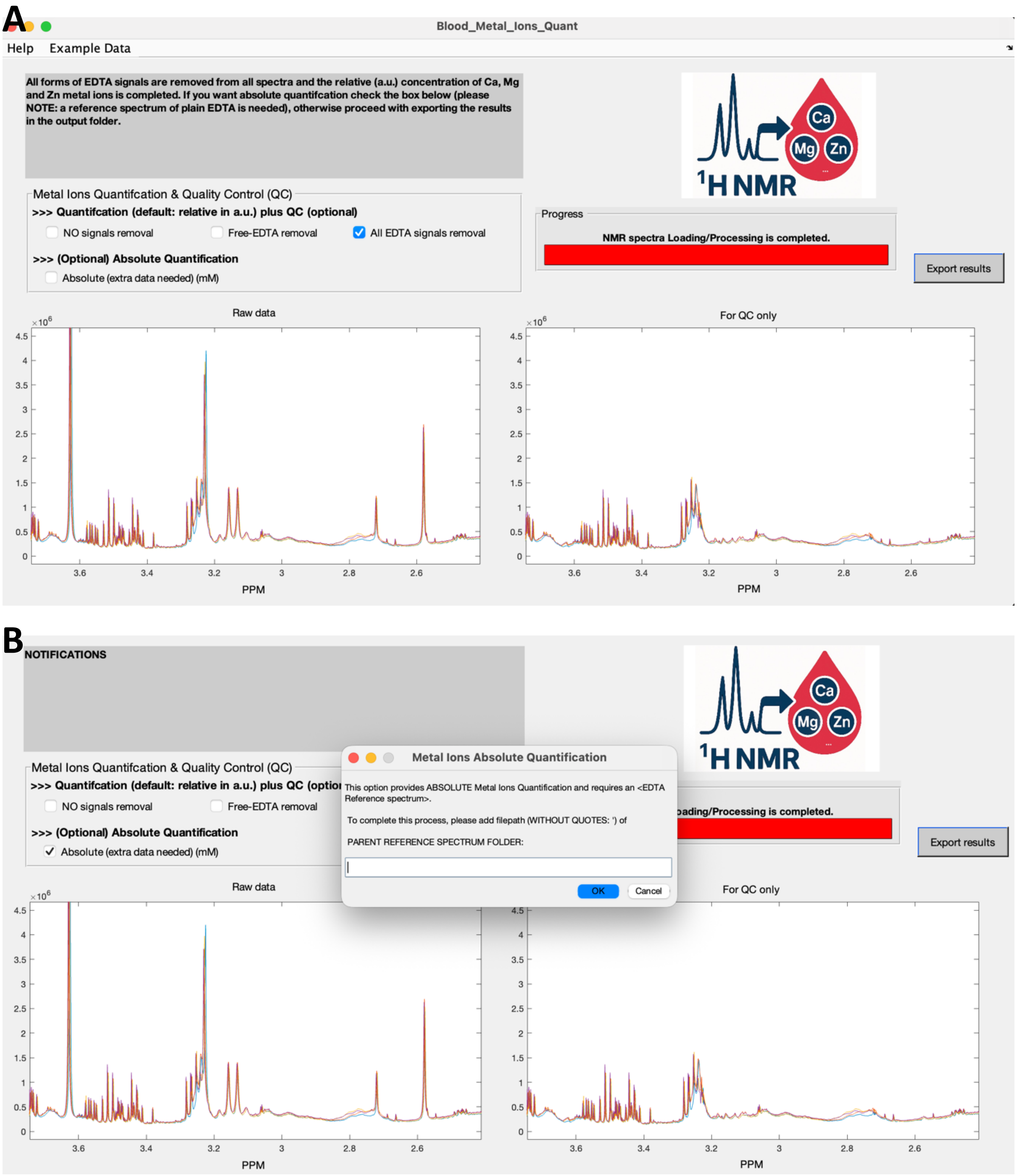

Figure S14. General overview of the software after loading several serum/plasma spectra containing EDTA. (**A**) When all "EDTA signals removal" option is selected, in the right panel all EDTA ^{1}H NMR signals are removed and the relative quantification of the Ca, Mg and Zn metal ions is performed (based upon the integrals of the characteristic singlets from their corresponding metal-EDTA complexes). Both QC'ed and integrals of the peaks can be exported to user's defined output folder in ".csv" format by simply pressing the button "Export results". (**B**) When "absolute quantification" option is selected, the user can upload an EDTA reference spectrum (prepared as indicated in the *Methods section* of the main manuscript) and re-export the results.

References

1. Wishart, D. S. *et al.* HMDB 3.0—The Human Metabolome Database in 2013. *Nucleic Acids Research* **41**, D801–D807 (2013).
2. Wishart, D. S. *et al.* HMDB 4.0: The human metabolome database for 2018. *Nucleic Acids Res.* **46**, D608–D617 (2018).
3. Yurekten, O. *et al.* MetaboLights: open data repository for metabolomics. *Nucleic Acids Res.* **52**, D640–D646 (2024).
4. Dona, A. C. *et al.* Precision High-Throughput Proton NMR Spectroscopy of Human Urine, Serum, and Plasma for Large-Scale Metabolic Phenotyping. *Anal. Chem.* **86**, 9887–9894 (2014).
5. Takis, P. G. *et al.* A Computationally Lightweight Algorithm for Deriving Reliable Metabolite Panel Measurements from 1D 1H NMR. *Anal. Chem.* **93**, 4995–5000 (2021).
6. Gegenschatz, S. A., Chiappini, F. A., Teglia, C. M., Muñoz de la Peña, A. & Goicoechea, H. C. Binding the gap between experiments, statistics, and method comparison: A tutorial for computing limits of detection and quantification in univariate calibration for complex samples. *Anal. Chim. Acta* **1209**, 339342 (2022).
7. Stebbing, J. *et al.* Comparison of phenomics and cfDNA in a large breast screening population: the Breast Screening and Monitoring Study (BSMS). *Oncogene* **42**, 825–832 (2023).
8. Harris, C. R. *et al.* Array programming with NumPy. *Nature* **585**, 357–362 (2020).
9. Virtanen, P. *et al.* SciPy 1.0: fundamental algorithms for scientific computing in Python. *Nat. Methods* **17**, 261–272 (2020).
10. Eilers, P. & Boelens, H. Baseline Correction with Asymmetric Least Squares Smoothing. *Unpubl. Manuscr* (2005).
11. Newville, M., Stensitzki, T., Allen, D. & Ingargiola, A. *LMFIT: Non-Linear Least-Square Minimization and Curve-Fitting for Python¶*. (2014). doi:10.5281/zenodo.11813.
12. Ida, T., Ando, M. & Toraya, H. Extended pseudo-Voigt function for approximating the Voigt profile. *J. Appl. Crystallogr.* **33**, 1311–1316 (2000).
